# Triphenylamine-Based Metal-Free Organic Dyes as Co-Sensitizers: Enhancing Dye-Sensitized Solar Cell Performance Through Innovative Molecular Design

**DOI:** 10.1007/s10895-025-04340-9

**Published:** 2025-05-16

**Authors:** Samar E. Mahmoud, Safa A. Badawy, Ahmed A. Fadda, Ehab Abdel-Latif, Mohamed R. Elmorsy

**Affiliations:** Department of Chemistry, Faculty of Science, Mansoura University, El-Gomhoria Street, Mansoura, 35516 Egypt

**Keywords:** Triphenylamine, D-*π*-A, Co-sensitizer, DFT, Photovoltaic performance

## Abstract

A series of novel dye-based D-π-A-type metal-free organic dyes was prepared *via* the Wittig reaction. Dyes **SAS-1-5** containing triphenylamine (**TPA**) as a strong electron donor was linked with an aryl ring as *a π*-conjugation spacer, cyanoacetamide, 2-(phenylsulfonyl)acetonitrile, and thiazolidine as the electron acceptors. Herein, we confirmed the chemical structures of the co-sensitized triphenylamine sensitizers using spectral analyses. In addition, their optical properties, electrochemical characteristics, and photovoltaic performances were estimated. Theoretical density functional theory (DFT) at the B3LYP/6-311G(d, p) level was used to elucidate the bridged effect on geometry. The **SAS-1-5** sensitizers showed absorption bands in a dimethylformamide (DMF) solution in the range of (450–590 nm) which led to an obvious enhancement in the visible harvesting ability. To improve the efficiency of the DSSCs, SAS-1-5 dyes were co-sensitized with the standard dye **N-719**. The power conversion efficiencies (*PCEs*) of **SAS-1-5** with **N-719** ranged from 7.39 to 9.12%. By employing **SAS-2** as a co-sensitizer, the DSSC system achieved an impressive 9.12% efficiency, which was 24% higher than that when using the **N-719** ruthenium complex dye. Furthermore, all TPA-based co-sensitizers (**SAS-1-5**) demonstrated considerable promise for improving photovoltaic performance.

**Supplementary Information:**

The online version contains supplementary material available at 10.1007/s10895-025-04340-9.

## Introduction

**Fig. 1:**
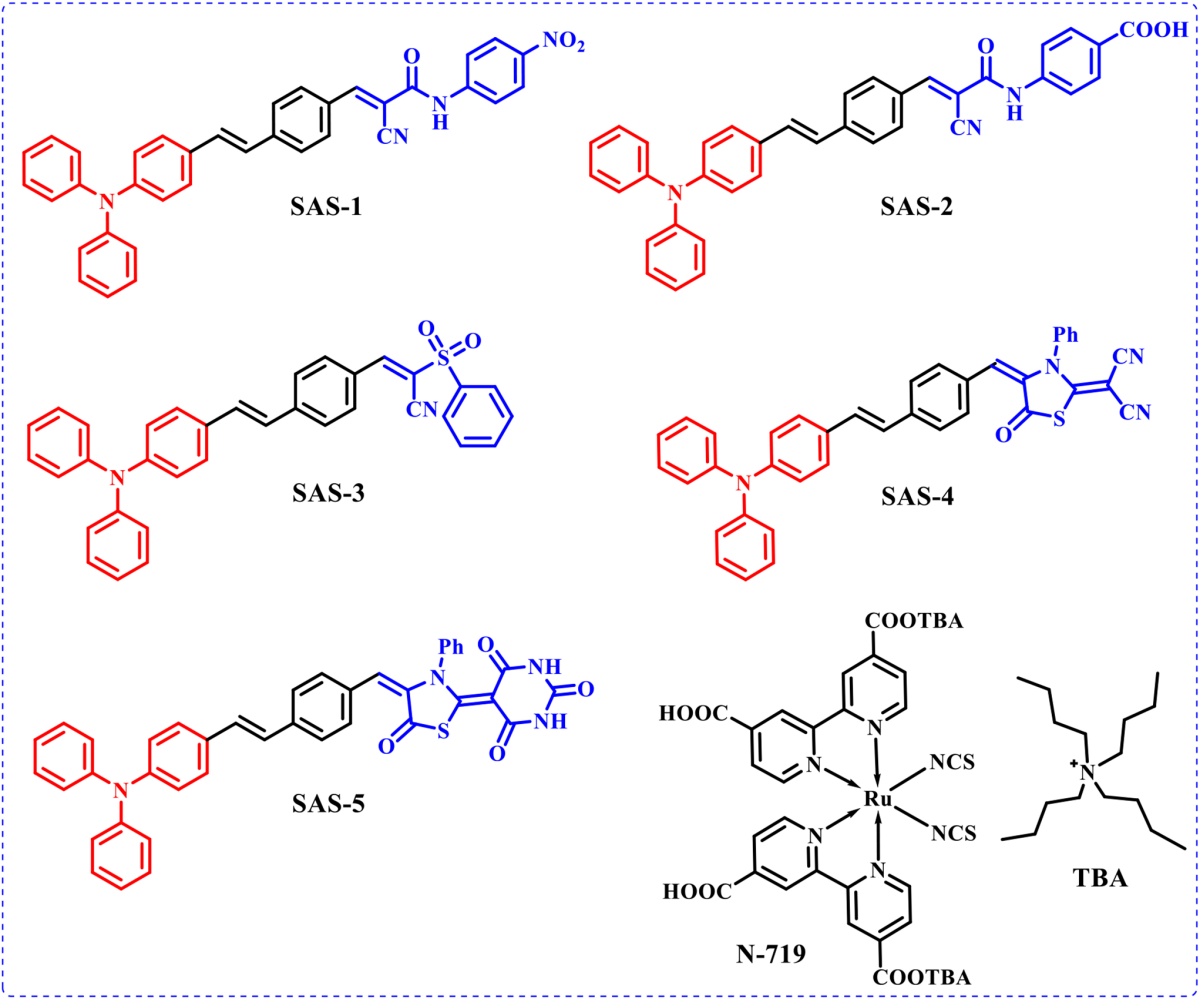
Molecular structures of triphenylamine co-sensitizers **SAS-1-5** with **N719**

Since their initial publication in 1991, Gratzel cell/dye-sensitized solar cells (DSSCs) have garnered considerable attention owing to their low-cost fabrication, high power-conversion efficiency, mutable shapes, colors, and transparency [1, 2]. They can be printed on lightweight substrates such as plastic or glass, as well as foils, providing spacious opportunities for integration into electronic devices, building materials, and novel markets where traditional silicon technology is inappropriate [3, 4]. DSSC have primary constituents: photoanodes, photosensitizers, counter electrodes, and electrolytes. As one of the most critical components, photosensitizers absorb photons of a special wavelength and inject photoexcited electrons into the conduction band (CB) of the nanoparticle surface (TiO_2_) [5]. They also exert a significant influence on the conversion efficiency and stability of cells [6]. In the past two decades, many species of photosensitizers have been developed, such as metal, organic, and natural dyes [7, 8]. Metal complexes (N3, N-719, and Black dyes) have attracted considerable research interest because of their broad absorption spectra, appropriate energy levels, and high efficiencies of over 11–13% [9–11]. However, metal complex dyes have several obstacles, such as metal toxicity and large sizes, which tend to leave empty spaces between the linked molecules on the surfaces of TiO_2_ [12]. Metal-free dyes are known to show molecular structure flexibility and spectral property tenability and gain recognition to provide much scope for building efficient and reliable components for DSSCs [13]. Diverse design strategies for dyes such as D-*π*-A, D-A-*π*-A, D-D-*π*-A, A-D-*π*-A, and A-*π*-D-*π*-A configurations have been reported [14, 15]. The three main components comprise a dye’s basic structure: the donor unit, which donates electrons to the sensitizer’s remaining parts; the *π*-bridge, which transfers electrons donated to the acceptor unit, which pulls the electrons passed on by the rest of the dye; and the acceptor moiety, which also has anchoring groups that can link the sensitizer to the semiconductor surface [16, 17]. Photovoltaic performance is enhanced when DSSCs are co-sensitized instead of using only one sensitizer [18]. This is due to the lack of dye aggregation and low charge recombination due to co-sensitizers filling the surface area between the larger sensitizers [19]. The triphenylamine (TPA) moiety is a discriminatory structural motif because of its strong electron-donating nature, superior hole-transporting capability, and good charge carrier mobility [20–22]. Herein, we incorporated a phenyl ring into triphenylamine units, and five dye-containing TPA were synthesized with different acceptor moieties. The synthesized dyes (D-*π*-A) included a strong donor unit (TPA), phenyl as *the π*-space, and diverse acceptor units (cyanoacetanilide, (phenylsulfonyl)acetonitrile, and thiazolidine-5-one) with various anchoring groups (CO, CN, and NH). **SAS-1-5** dyes were used as co-sensitizers with the Ru(II) complex (**N-179**) to improve the performance of DSSCs. The optical behavior was investigated using UV-Vis absorption and was found to be in the range of (450–590 nm). In addition to the electrochemical and photovoltaic properties, the electrochemical impedance spectra of the designed co-sensitizers were studied and compared to **of those N-719**. Based on these research findings, the co-sensitizer (**SAS-2** + **N-719**) produced a higher *PCE* (9.12%) than **N-719** (7.33%). This dye **SAS-2** is an outstanding choice for DSSC applications. Moreover, it provided the highest *J*_*SC*_ of 24.27 mA/cm² and *V*_*OC*_ = 0.69 V. The chemical structures of the organic dye **SAS-1-5** and the reference dye **N-719** are shown in Fig. 1.

## Experimental Section

The synthesis and characterization of all synthesized compounds were explained in detail in the supplementary file in (Figure **S1-28**).

### Results and Discussions

#### Synthesis

**Scheme 1:**
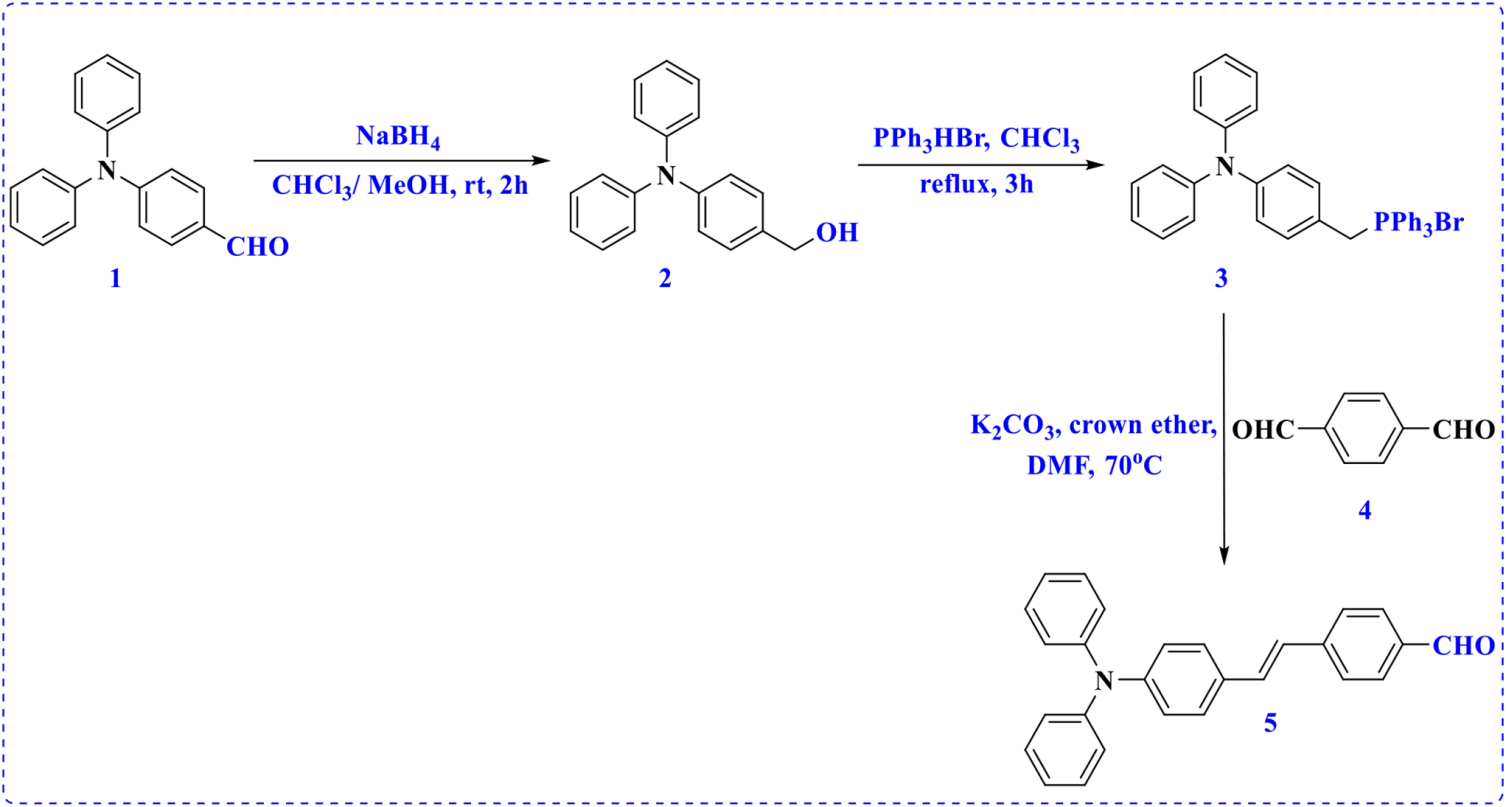
Synthesis of 4-(4-(diphenylamino)styryl)benzaldehyde (**5**)

The reduction of 4-(diphenylamino)benzaldehyde (**1**) [23] was achieved as previously described [24]. Hydroxyl methyl compound **2** was reacted with (λ^2^-bromaneyl)triphenyl- λ^4^-phosphane to produce phosphonium salt **3** [25]. The synthetic pathway for 4-(4-(diphenylamino)styryl)benzaldehyde (**5**) occurred under Wittig reaction conditions between compound **3** and terephthalaldehyde (**4**), as shown in Scheme 1. The molecular structure of styrylbenzaldehyde derivative **5** was confirmed using spectral data. The ^1^H NMR spectrum displayed the distinctive doublet signal for two protons of the vinylic moiety at *δ* 7.19 and 7.41 ppm and the protons of phenyl rings were observed as doublet, triplet, and multiplet signals in the range of 6.94–7.87 ppm. In addition, the proton of the formyl group is observed as a singlet at *δ* 9.95 ppm. In the ^13^C NMR spectrum, the characteristic signal of the carbon atom of the aldehyde group was observed in the downfield area at *δ* 192.29 ppm.

**Scheme 2:**
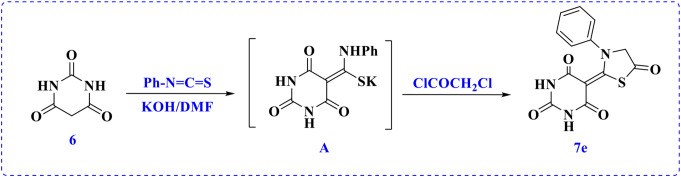
Synthesis of 5-(5-oxo-3-phenylthiazolidin-2-ylidene) pyrimidine-2,4,6(1*H*,3*H*,5*H*)-trione (**7e**)

Styrylbenzaldehyde compound **5** was combined with different acceptors, such as 2-cyanoacetamide derivatives **7a** and **7b** [26], 2-(phenylsulfonyl)acetonitrile (**7c**), and thiazolidine-5-one derivative **7d** [27]. For the new acceptor, 5-(5-oxo-3-phenylthiazolidin-2-ylidene)pyrimidine-2,4,6(1*H*,3*H*,5*H*)-trione (**7e**) was synthesized by adding the active methylene of barbituric acid to phenyl isothiocyanate in DMF as a solvent and potassium hydroxide (KOH) as the basic catalyst to form the non-isolated sulfide intermediate (**A**). The sulfide salt underwent in situ addition of chloroacetyl chloride to furnish the corresponding thiazolidin-5-one derivative **7e**, as described in Scheme 2. The structure of this thiazolidine was proven using spectral methods. For instance, the infrared spectrum showed absorption peaks at 3464 and 3392 cm^− 1^ for the amino group and other peaks at 1708 and 1644 cm^− 1^ due to the C = O (thiazolidine) and C = O (amidic) groups, respectively. In the ^1^H NMR spectrum, the protons of the methylene group of the thiazolidine ring appeared as singlets at *δ* 4.17 ppm. The five aromatic protons were found as doublets and triplets at *δ* 7.32–7.44 ppm. The two amino groups appeared as a broad singlet at 11.65 and 12.38 ppm.

**Scheme 3:**
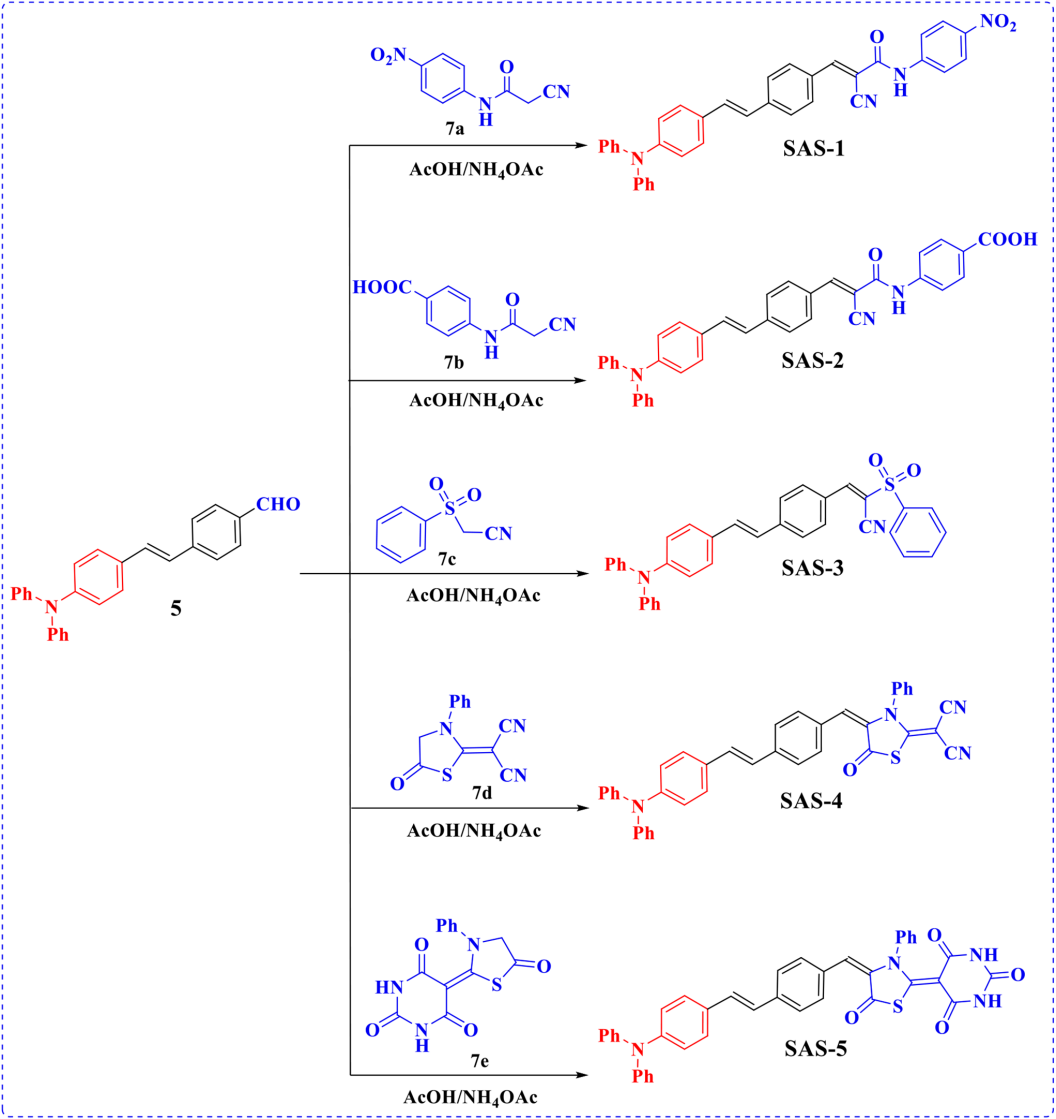
Synthetic route of triphenylamine-based sensitizers **SAS-1-5**

The five targeted metal-free dyes based on the TPA moiety **SAS-1-5** were obtained in yields of 56%, 49%, 51%, 53%, and 59% via the Knoevenagel condensation reaction of precursor 4-(4-(diphenylamino)styryl)benzaldehyde (**5**) with two cyanoacetamide derivatives **7a** and **7b**, 2-(phenylsulfonyl)acrylonitrile (**7c**), and two thiazolidine derivatives **7d** and **7e**, respectively, in the presence of ammonium acetate as a good catalyst, as described in Scheme 3. The molecular structure of the new chromophore, SAS-1-5, was confirmed through spectral data and elemental investigation. The IR spectrum of sensitizer **SAS-1** displayed characteristic absorption peaks of cyano (C ≡ N) at 2218 cm^− 1^ and carbonyl (C = O) at 1689 cm^− 1^. For ^1^H NMR spectrum gave doublet signals at *δ* 7.19 and 7.42 ppm that could be attributed to protons of the vinylic group with (*J* = 16.00 Hz), singlet signals at *δ* 8.27 and 10.92 ppm for olefinic proton and N-H group. Furthermore, the IR spectra of the co-sensitizer SAS-2 revealed distinct absorption bands due to amino and hydroxyl groups at 3328 and 2548 cm^− 1^, respectively. In addition, the nitrile group, carbonyl group, and olefinic double bond (sp^2^ hybridization) resonated at 2221, 1694, and 1581 cm^− 1^, respectively. In the ^1^H NMR spectrum, two protons of the vinylic unit exhibited doublet signals at *δ* 7.19 and 7.41 ppm with *J* = 16.00 Hz. The three protons of the olefinic, amino, and carboxylic groups appeared as singlet signals in the high-frequency region at 8.25, 10.65, and 12.44, respectively. Moreover, **SAS-3** gave special peaks at 1327 and 1314 cm^− 1^ for the sulfonyl group (SO_2_). The ^1^H NMR spectrum of chromophore **SAS-3** displayed two doublet signals for the vinylic protons at *δ* 7.19 and 7.46 ppm. The proton of the other olefinic group was observed as a singlet at *δ* 8.49 ppm. Its ^13^C NMR spectrum displayed a distinctive signal for the cyano group at 113.69 ppm. Otherwise, the ^13^C NMR spectrum of **SAS-4** revealed distinctive signals at *δ* 113.96 and 116.56 ppm for two nitrile groups. The signals of aromatic carbons were observed in the range 122.43-165.86 ppm. Furthermore, the carbon atom of the carbonyl group in the thiazolidine-5-one ring was observed at *δ* 167.02 ppm. The skeleton of the final dye, SAS-5, was determined by spectroscopy; for example, the mass spectrum displayed a molecular ion peak at m/z = 660 (32.39%).

## Photophysical Properties

**Fig. 2:**
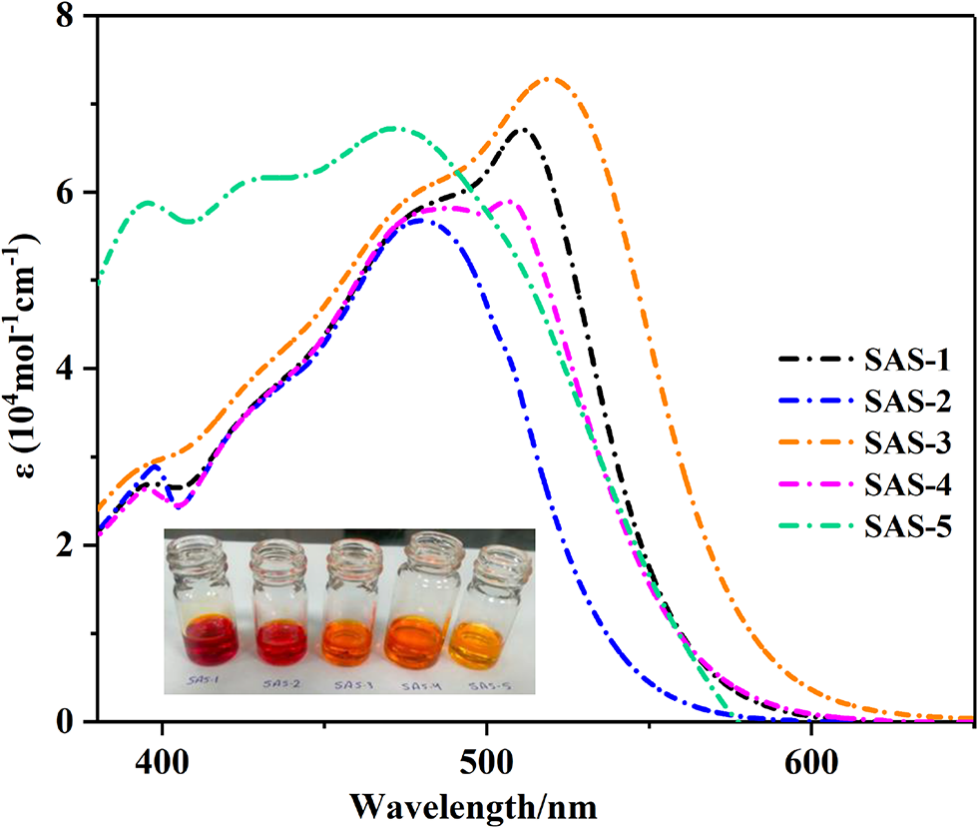
UV-visible. absorption of dyes **SAS-1-5**

The chromophore’s **SAS-1-5**’s UV-Vis absorption spectra of all chromophores in DMF (2 × 10^− 5^ M) are shown in Fig. 2, and Table 1 presents the relevant findings and unique spectral data.

**Table 1:** Absorption results for triphenylamine dyes **SAS-1-5**

Sensitizer	λ_max_ (nm)	ε (10^4^ M^− 1^ cm^− 1^)	λ_onset_ / nm	Experimental E_0 − 0_ (eV)
SAS-1	396, 511	2.67, 6.70	571	2.17
SAS-2	397, 480	2.87, 5.67	546	2.27
SAS-3	394, 519	2.87, 7.27	584	2.12
SAS-4	395, 506	2.63, 5.89	562	2.20
SAS-5	395, 471	5.87, 6.72	566	2.19

The absorption spectrum of the reference dye **N-719** is shown in Fig. S1 exhibit two distinct absorption bands for each sensitizer. These bands, observed in the shorter wavelength range of 390–430 nm, can be attributed to π-π* electronic transitions within the chromophore, specifically localized in the donor unit (TPA) and the *π*-conjugated phenyl system. An intramolecular charge transfer (ICT) process occurs from the triphenylamine donor to its corresponding anchoring units, as evidenced by the absorption peaks with maximum wavelengths ranging from 450 to 590 nm. Furthermore, the onset of the UV-visible absorption spectrum provides the basis for calculating the energy gap (*E*_*0 − 0*_) [28]. *E*_*0 − 0*_ was reduced by the addition of a phenyl ring as *a π*-spacer, which promoted visible light-harvesting capabilities [29], **SAS-3 < SAS-1 < SAS-5 < SAS-4 < SAS-2**. Furthermore, the molar extinction coefficients (*ε*) of the above sensitizers appeared at 6.70, 5.67, 7.27, 5.89, and 6.72 (10^4^ M ^− 1^ cm ^− 1^), which are related to their lowest energy gaps, which are noticeably higher than that of the reported value of **N-719** dye (*ε* = 1.08 × 10^4^ M ^− 1^ cm^− 1^), affirming their good light harvesting [30]. The absorption spectrum of **SAS-3** exhibited a pronounced red shift and a broader profile than those of the other dyes. This can be attributed not only to the presence of the strongly electron-withdrawing sulfonyl group, which enhances the ICT character and reduces the energy gap, but also to the increased molecular planarity and extended π-conjugation. These structural features promote better orbital overlap and allow for greater delocalization of the excited state, resulting in a broadened absorption band. Moreover, the interaction between the sulfonyl group and the conjugated π-system may facilitate multiple closely spaced electronic transitions, which collectively contribute to the broader spectral envelope of the observed photophysical behavior of **SAS-3** [31]. Further insight revealed that **SAS-1** and **SAS-3** were more red-shifted than **SAS-2**, **SAS-4**,** and SAS-5** in their absorption spectra, which can be ascribed to the extension of the conjugation system within molecules and the presence of electron-withdrawing groups across **SAS-1** and **SAS-3** [32]. **SAS-2** with a cyanoacrylamide moiety as the acceptor showed a hypsochromic shift compared to all the produced sensitizers, indicating that cyanoacrylamide attached to a carboxylic group has a lower electron withdrawal ability, which makes it unsuitable for light absorption.

**Fig. 3:**
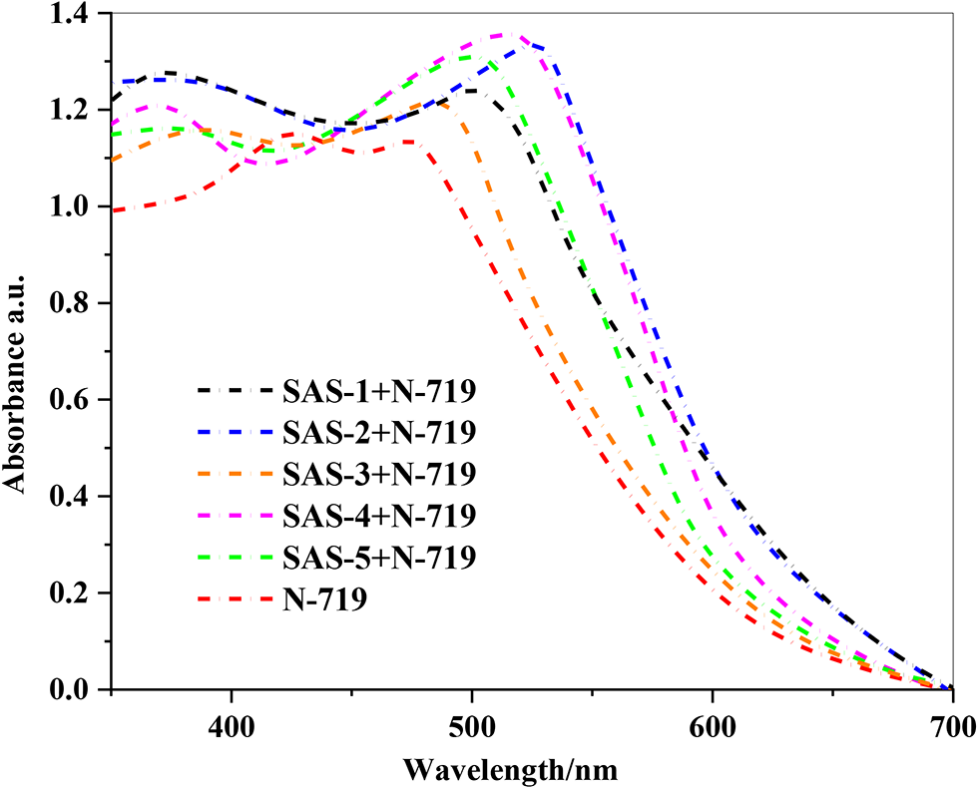
Absorption spectra sensitized and co-sensitized triphenylamine sensitizers **SAS-1-5** with **N-719** adsorbed on nonporous TiO_2_

A comparison of the absorption spectra of the dyes shows that the co-sensitizers (**SAS-1-5**) along with **N-719** exhibit a broader spectral range in thin-film form, which is advantageous for promoting the photocurrent density (*J*_*SC*_) of the newly developed dyes. Moreover, their adsorption onto the TiO_2_ surfaces induced a red shift due to *J*-aggregation (Fig. 3) [33, 34]. These shifts in the UV-Vis spectra were likely the result of strong interactions between the carboxylate and various acceptors of the dyes and the TiO_2_ surface, leading to the formation of *J*-aggregates. Upon analyzing the absorption spectra in Fig. 3, all sensitizers (**SAS-1-5 + N-719**) exhibited higher absorbance values than **N-719** alone, indicating higher dye loading on the TiO_2_ surface. This enhanced dye loading is critical for achieving improved light-harvesting efficiency, which directly correlates with the higher (*J*_*SC*_) in (DSSCs). The wider and red-shifted absorption profile of the (**SAS-2 + N-719**) system suggests a higher dye loading and more extensive absorption than other co-sensitizers. This enhanced performance can be attributed to **the SAS-2**, 4-carboxylcyanoacetamide moiety, which interacts more effectively with TiO_2_, facilitating more efficient light capture, and consequently, higher photocurrent generation (*J*_*SC*_).

## Theoretical Calculation for SAS-1-5 Dyes

**Fig. 4:**
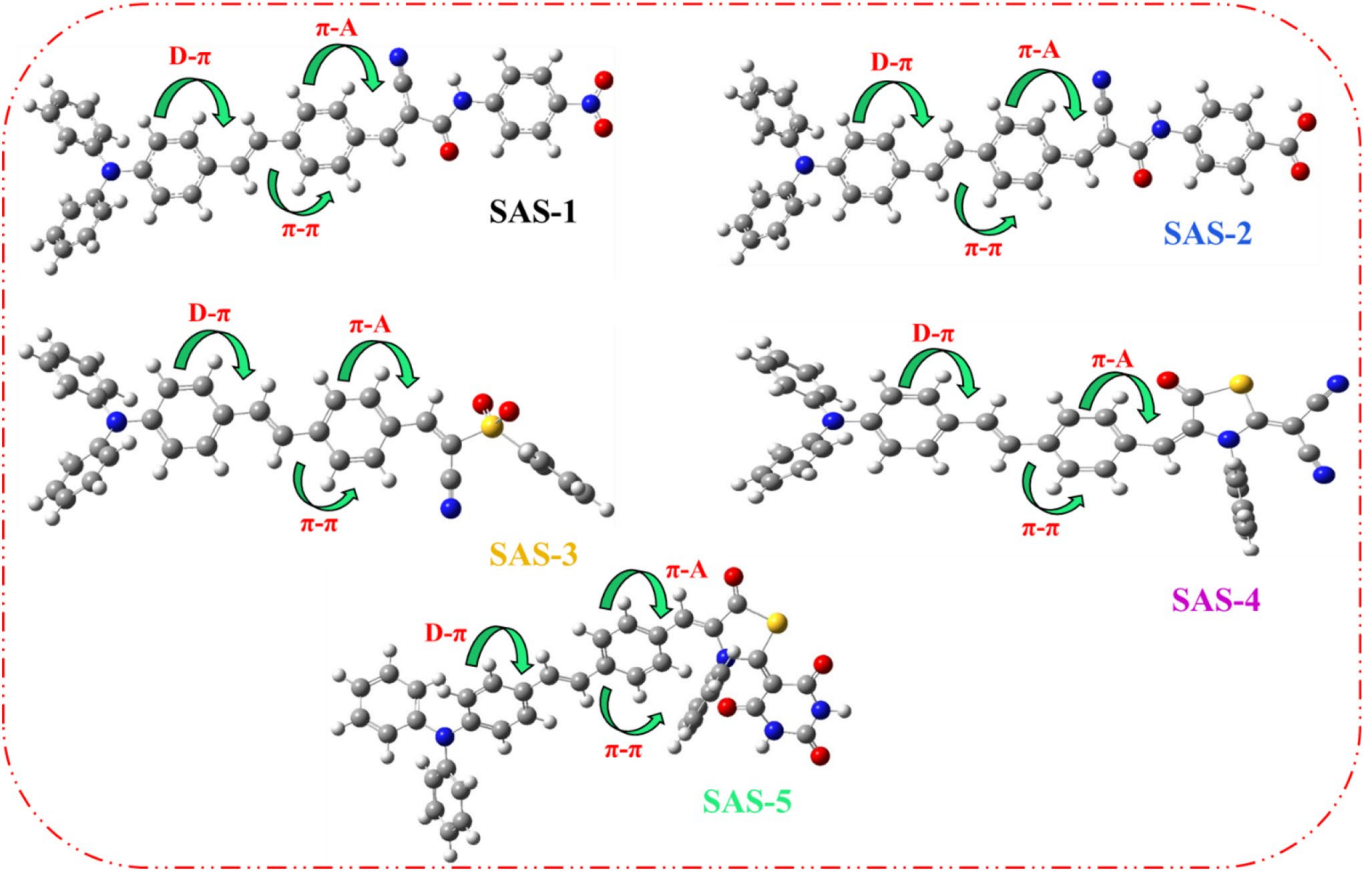
Optimized structures of the TPA sensitizers **SAS-1-5**

Theoretical calculations for chromophores (**SAS-1-5**) were performed to determine how the *π*-spacer and diverse acceptor units, cyanoacetamide, sulfonyl acetonitrile, and thiazolidine derivatives, affect the geometry of the targeted dyes and the performance of DSSCs. The Gaussian 09 program used the 6-311G(d, p) basis set [35] and B3LYP functional to perform the optimized ground state (Fig. 4).

The bond lengths and dihedral angles of the sensitizers are listed in Table 2. The dihedral angle between the π-conjugated bridge and acceptor in **SAS-1** to **SAS-5** plays a crucial role in facilitating intramolecular charge transfer (ICT). According to the DFT-optimized geometries, **SAS-1**–**SAS-4** exhibited nearly planar configurations with dihedral angles close to 180° (ranging from 179.39° to 179.90°), promoting strong π-conjugation and efficient orbital overlap between the donor and acceptor segments. This structural planarity enhances ICT, leading to improved electron injection efficiency into TiO_2_, red-shifted absorption, and improved light-harvesting properties. In contrast, **SAS-5** exhibited a relatively lower dihedral angle of 165.53°, indicating some torsional distortion that may reduce conjugation and hinder optimal charge transfer. The dihedral angle closer to 180° significantly supports charge delocalization and ICT efficiency, highlighting the importance of molecular planarity in optimizing the photovoltaic performance of D-π-A dyes [36]. The bond lengths for **SAS-1-5** were measured at 1.45–1.46 Å for the D-*π* linkage and 1.44 Å for the *π*-A linkage.

**Table 2:** Calculated dihedral and bond lengths of dyes **SAS-1-5**

Sensitizers	Dihedral angles (°)			Bond lengths (Å)		
	D-π (°)	π-π (°)	π-A (°)	D-π (Å)	π-π (Å)	π-A (Å)
SAS-1	175.22	176.62	179.39	1.45	1.45	1.44
SAS-2	175.41	176.64	179.51	1.45	1.45	1.44
SAS-3	179.25	179.53	179.87	1.46	1.46	1.44
SAS-4	178.35	179.16	179.90	1.45	1.45	1.44
SAS-5	175.43	175.84	165.53	1.46	1.46	1.44

## Natural Bond Orbital Analysis (NBO) for TPA-sensitizers SAS-1-5

The main objective of population analysis is to utilize Natural Bond Orbital (NBO) analysis, which illustrates the charge distribution and the electron transfer process from the donor (occupied) orbital to the acceptor (unoccupied) orbital within the **D-*π*-A** system [37]. The NBO Analysis’s computations were carried out using B3LYP functional and 6-311G(d, p) basis sets [38]. This method is highly effective for exploring both inter- and intra-molecular interactions and offers a reliable framework for studying conjugated systems in the sensitizers **SAS-1-5** [39]. For target sensitizers, greater energy values of E^(2)^ (Kcal/mol) hyper-conjugative interactions offer a more outright interaction between a strong donor (TPA) and diverse acceptor units (CN, NO_2_, COOH, and thiazolidine-5-one) indicating the higher ability for donation from donor to acceptors. The highest stabilization energies **E**^**(2)**^ of **SAS-1-5** and various transitions which originated from D-*π*, *π*-*π*, and *π*-A were introduced in Table 3.

**Table 3:** Natural bond orbital analysis (NBO) of dyes **SAS-1-5**

Sensitizers	Transitions	Stabilization energy E^(2)^ (Kcal/mol)
SAS-1	π*(C2-C15) → π*(C18-C19) π*(C22-C23) → π*(C20-C21) π*(C30-O32) → π*(C28-C29)	248.39 83.58 35.88
SAS-2	π*(C2-C15) → π*(C18-C19) π*(C22-C23) → π*(C20-C21) π*(C41-O42) → π*(C35-C36)	250.67 84.50 208.87
SAS-3	π*(C16-C17) → π*(C20-C21) π*(C22-C23) → π*(C20-C21) π*(S30-O38) → π*(S30-O37) π*(C31-O36) → π*(C32-C33)	149.70 83.07 45.21 263.19
SAS-4	π*(C16-C17) → π*(C20-C21) π*(C22-C23) → π*(C20-C21) π*(C30-O34) → π*(C28-C29)	129.00 82.06 47.75
SAS-5	π*(C11-C12) → π*(C20-C21) π*(C22-C27) → π*(C20-C21) π*(C39-O43) → π*(C31-C34)	124.51 113.21 239.43

### Theoretical Chemical Parameter for TPA-sensitizers SAS-1-5

There are different theories for predicting several physicochemical parameters introduced by B3LYP/6-311G(d, p) method. The chemical reactivity factors of the sensitizers **SAS-1-5** could be evaluated with the framework of Koopmans’ theory (Eqs. 1–4) [40] and presented in Table 4.

1 $$\:\varvec{I}\varvec{P}=-{\varvec{E}}_{\varvec{H}\varvec{O}\varvec{M}\varvec{O}}$$

2 $$\:\varvec{E}\varvec{A}=-{\varvec{E}}_{\varvec{L}\varvec{U}\varvec{M}\varvec{O}}$$

***η =***$$\:\:\left(\frac{{\varvec{E}}_{\varvec{L}\varvec{U}\varvec{M}\varvec{O}}-{\varvec{E}}_{\varvec{H}\varvec{O}\varvec{M}\varvec{O}}}{2}\right)$$             (3)

4 $$\:\varvec{s}=\frac{1}{\varvec{\eta\:}}$$

The energy gap (*E*_*g*_) was calculated by the difference among energies of the highest occupied molecular orbital (HOMO) and the lowest unoccupied molecular orbital (LUMO) levels. *E*_*g*_ values for sensitizers **SAS-1-5** decreased in the following sequence: **SAS-3** < **SAS-5** < **SAS-1** < **SAS-4** < **SAS-2**. Sensitizer **SAS-3** with 2-(phenylsulfonyl)acetonitrile as an acceptor showed the smallest energy gap value. The smallest value *E*_*g*_ of the **SAS-3** facilitated excitation between the HOMO and LUMO and confirmed its highest stability. The hardness (*η*) and softness (*s*) are beneficial variables to help know the system’s reactivity [41]. The stability and reactivity of molecules are correlated with their hardness [42]. Moreover, the global softness for **SAS-1-5** sensitizers ranged from 0.87 to 0.93 eV. On the other hand, several key photovoltaic parameters influence the performance of DSSC, such as the driving forces of the electron injection (**ΔG**_**inj.**_), the electron regeneration (**ΔG**_**reg**_), and the electron recombination (**ΔG**_**rec**_.). These forces were studied as shown in Eqs. (5–7) [43].

5 $$\:\varDelta\:\varvec{G}\varvec{i}\varvec{n}\varvec{j}\:\left(\varvec{e}\varvec{V}\right)=\:{\varvec{E}}_{\varvec{O}\varvec{X}}^{{\varvec{d}\varvec{y}\varvec{e}}^{\varvec{*}}}-\:{\varvec{E}}_{\varvec{C}\varvec{B}}$$

6 $$\:\varDelta\:\varvec{G}\varvec{r}\varvec{e}\varvec{g}\:\left(\varvec{e}\varvec{V}\right)=\:{\varvec{E}}_{\varvec{O}\varvec{X}}^{\varvec{d}\varvec{y}\varvec{e}}-\:{\varvec{E}}_{\varvec{r}\varvec{e}\varvec{d}\varvec{o}\varvec{x}}$$

7 $$\:\varDelta\:\varvec{G}\varvec{r}\varvec{e}\varvec{c}.\:\left(\varvec{e}\varvec{V}\right)=\:{\varvec{E}}_{\varvec{O}\varvec{X}}^{\varvec{d}\varvec{y}\varvec{e}}-\:{\varvec{E}}_{\varvec{C}\varvec{B}}$$

The **ΔG**_**inj.**_ for dyes **SAS-1-5** was estimated as given in Eq. (5) and **ΔG**_**inj.**_**=** -1.07, -1.26, -1.04, -1.12, and − 1.17 eV, respectively. All dyes exhibited negative values for **ΔG**_**inj**_ that easily inject electrons into the CB of TiO_2_. The **Δ**G_inj_. of the TPA dyes decline in the following order: **SAS-2** > **SAS-5** > **SAS-4** > **SAS-1** > **SAS-3**. Importantly, **SAS-2** displayed the greatest value compared to other TPA-dyes, which due to acceptor and anchoring moieties (CO, CN, NH, and COOH) may improve electron injection driving power. For the **SAS-1-5** sensitizers, ΔG_rec_ values ranged from 1.01 to 1.12 eV, which may stem from the varying acceptor units’ ability to partially suppress charge recombination.

**Table 4:** Calculated parameters for TPA-sensitizers **SAS-1-5**

Dye	HOMO	LUMO	E_0 − 0_	IP	EA	η	s	∆G_inj._ (eV)	∆G_reg._ (eV)	∆G_rec._ (eV)
SAS-1	-5.32	-3.13	2.19	5.32	3.13	1.09	0.92	-1.07	0.12	1.12
SAS-2	-5.23	-2.94	2.29	5.23	2.94	1.14	0.87	-1.26	0.03	1.03
SAS-3	-5.31	-3.16	2.15	5.31	3.16	1.07	0.93	-1.04	0.11	1.11
SAS-4	-5.30	-3.08	2.22	5.30	3.08	1.11	0.90	-1.12	0.10	1.10
SAS-5	-5.21	-3.03	2.18	5.21	3.03	1.09	0.92	-1.17	0.01	1.01

## Electrochemical Properties for Sensitizers SAS-1-5

**Fig. 5:**
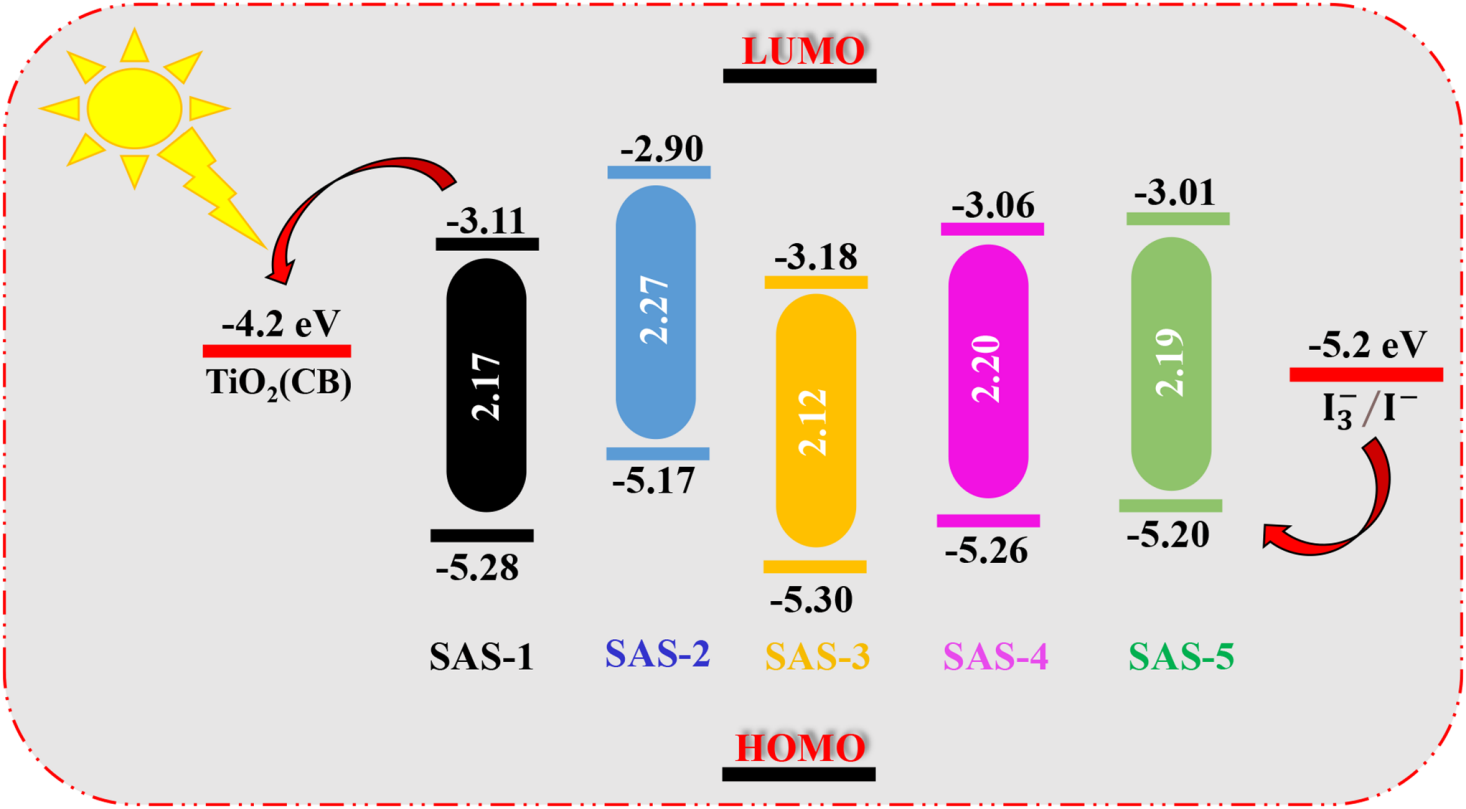
Experimental HOMO_S_ and LUMO_S_ levels for TPA-co-sensitizers **SAS-1-5**

Cyclic voltammetry measurements were performed to assess essential electronic factors, enabling the evaluation of the suitability of TPA-based sensitizers (**SAS-1-5**) for use in (DSSC) applications [44]. The observed voltammograms, HOMO-LUMO energy levels, and calculated band gaps are summarized in Fig. 5. The practical ground state oxidation potential (GSOP) energy levels, which represent the HOMO energy levels, range from − 5.17 to -5.30 eV. These values are well below the redox potential of redox pair (-5.20 eV), ensuring efficient dye regeneration after electron injection [45]. The excited state oxidation potential (ESOP) values for **SAS-1-5**, calculated using the Eq. (8) below, range from − 2.90 to -3.18 eV, indicating that all dyes have sufficiently negative values relative to the TiO_2_ conduction band (-4.20 eV), facilitating efficient electron injection.

***ESOP = - [(***$$\:{E}_{onset}^{oxd}$$***+4.7)-E***_***0–0***_***] eV***             (8).

The experimental optical band gaps (*E*_*0 − 0*_) ranged from 2.12 eV to 2.27 eV for sensitizers, aligning well with theoretical predictions and reflecting the dyes’ light-harvesting capabilities. **SAS-2** exhibits the highest LUMO level (-2.90 eV) among the sensitizers, making it the most efficient candidate for electron injection into the TiO_2_ conduction band (-4.20 eV). This higher LUMO value ensures a strong driving force for electron transfer, improving the overall efficiency of the dye sensitizer. **SAS-2** emerges as the best candidate for DSSCs due to its optimal combination of the highest LUMO value and favorable energy alignment for both electron injection and regeneration, making it the most promising candidate for DSSC applications [46].

## Molecular Modeling for SAS-1-5 Sensitizers

**Fig. 6:**
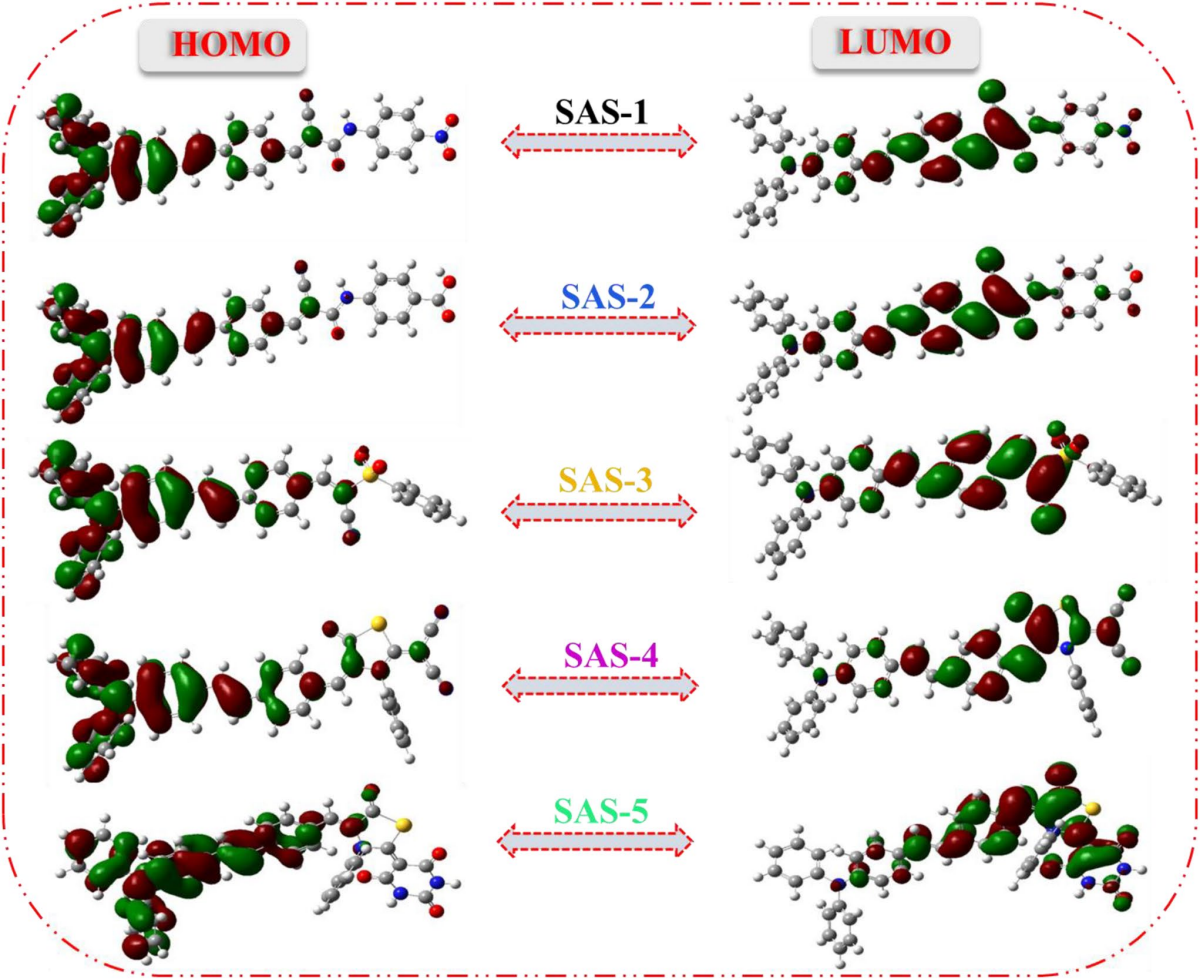
Optimized geometry structures for sensitizers **SAS-1-5**

In the present study, density functional theory (DFT) and time-dependent density functional theory (TD-DFT) were done for the dyes **SAS-1-5** at B3LYP hybrid method with a basis set at 6-311G(d, p) run by the Gaussian 09 software to detect the molecular geometry and electron distribution in the energy levels [47, 48]. One of the most important characteristics for studying charge separation is the delocalization of charges from the donor’s HOMO levels to the acceptor’s LUMO levels. For all **s**ensitizers, the electron density of the HOMO level was mainly distributed on triphenylamine (donor species) and phenyl unit (*π*-spacer). While the LUMOs for **SAS-1** and **SAS-2** localized over the cyanoacetamide area (CN and carbonyl) but not extended to COOH and NO_2_ segments which reflected the poor efficiency of these sensitizers. Noticeably, the introduction of (phenylsulphonyl)acetonitrile into the **SAS-3** dye shifted the distribution of electrons to the acceptor section, especially the cyano and sulfonyl regions. The thiazolidine-5-one ring for **SAS-4** and **SAS-5** enhances electrons flow from the triphenylamine and *π*-spacer to the acceptor portions that are represented in the (C ≡ N and C = O) groups. According to Fig. 6, the electron injection had significantly enhanced from the excited sensitizers to the semiconductor surface (TiO_2_).

## Molecular Electrostatic Potential (MEP) of Sensitizers SAS-1-5

**Fig. 7:**
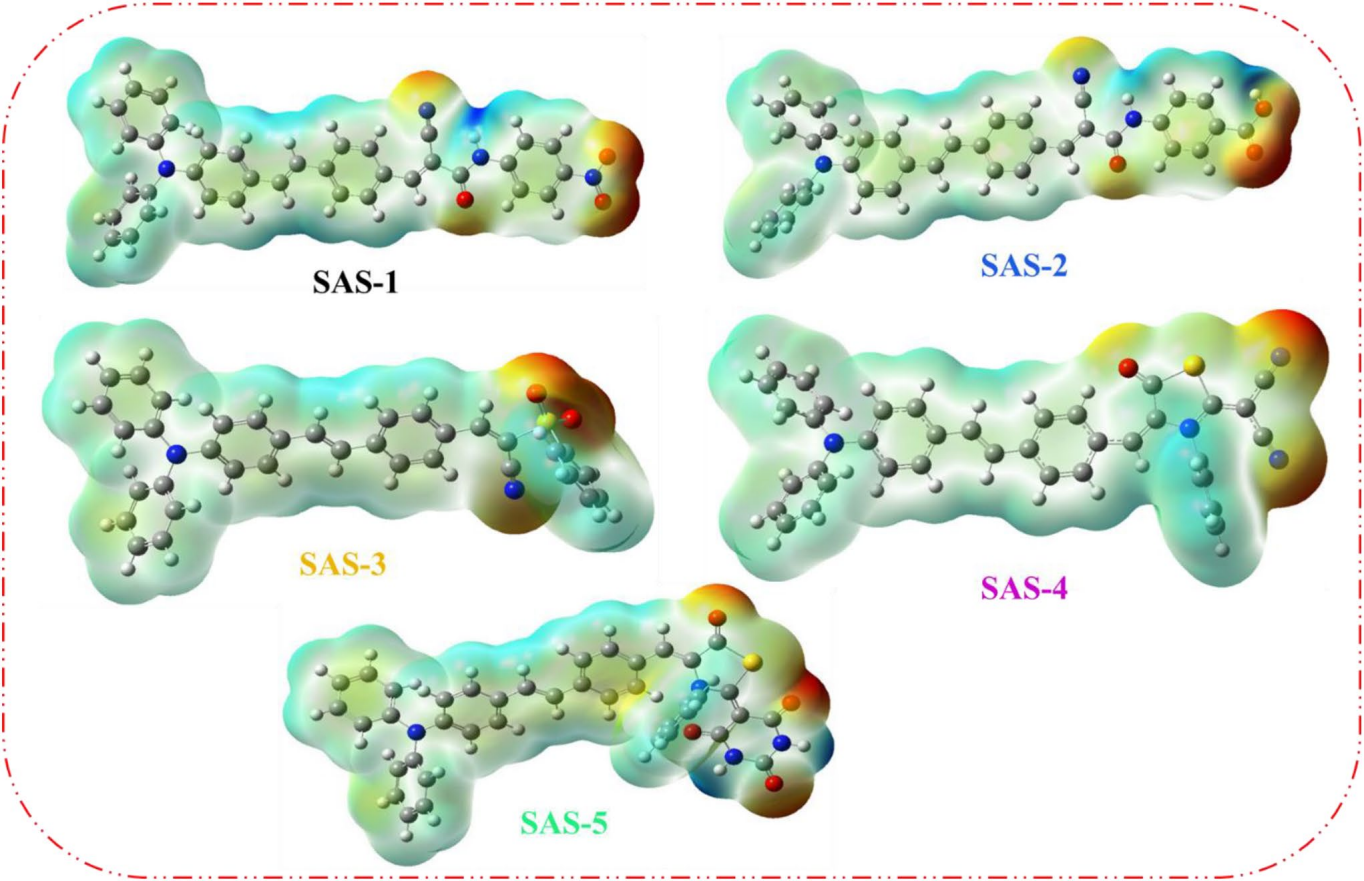
Molecular electronic potential diagram (MEP) of sensitizers **SAS-1-5**

The molecular electrostatic potential (MEP) analysis of the **SAS-1** to **SAS-5** sensitizers offers valuable insight into their electronic distribution and interaction potential with semiconductor surfaces [49]. The MEP maps, computed at the B3LYP/6-311G(d, p) level, reveal that the most electron-rich regions (red zones) are localized around the anchoring and electron-accepting moieties, including cyano (-CN), carbonyl (C = O), sulfonyl (-SO₂), nitro (-NO₂), and carboxyl (–COOH) groups as shown in Fig. 7. These areas represent highly nucleophilic sites that are likely to serve as reactive centers for binding with the surface of TiO_2_ via coordination or hydrogen bonding. Conversely, the electron-deficient zones (blue regions) are primarily distributed over the triphenylamine (TPA) donor units, consistent with their role in facilitating hole transport during the charge separation process. Notably, the spatial extent and intensity of the negative electrostatic potential directly influence the anchoring capability of the dye molecules on the TiO_2_ surface, which in turn affects dye loading, electron injection efficiency, and overall device performance. For instance, **SAS-2** and **SAS-5** display more extensive and intense negative potential regions near multiple anchoring sites (COOH, CN, and NH), suggesting stronger dye–semiconductor interactions. This correlates well with their superior photovoltaic metrics, including higher (*J*_*SC*_) and (*PCE).* Therefore, MEP mapping not only elucidates the charge distribution within these D-π-A systems but also serves as a predictive tool for evaluating dye adsorption strength and its consequent impact on DSSC efficiency.

### Photovoltaic Device Characterizations

**Fig. 8:**
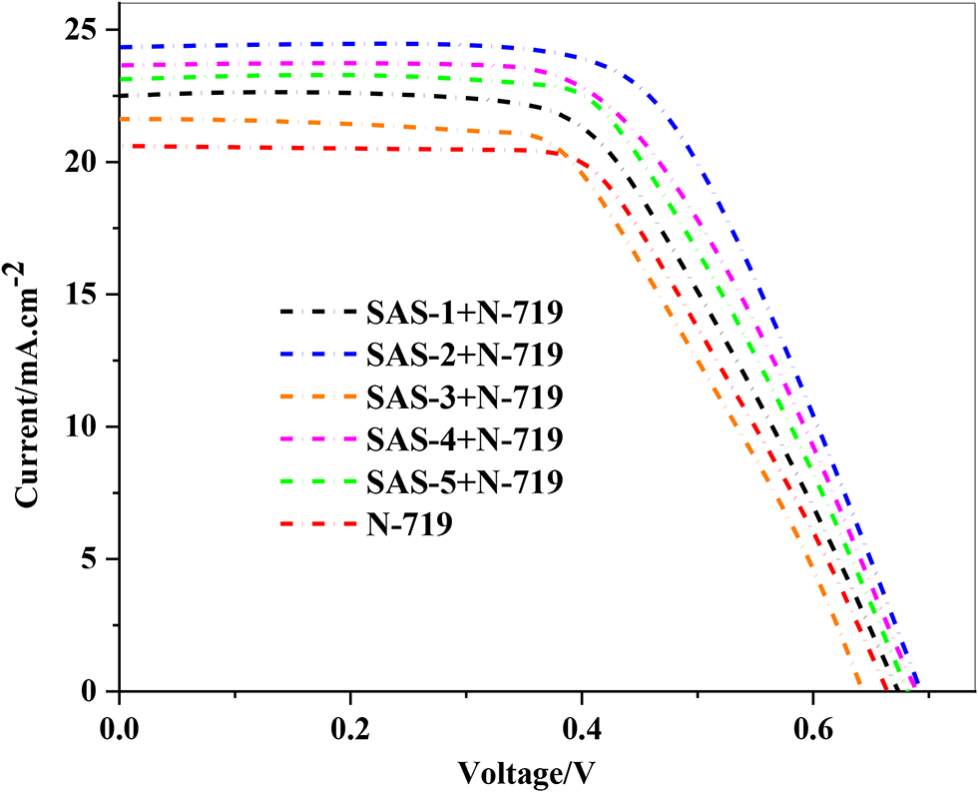
*J-V* curves of sensitized and cocktail co-sensitized triphrnylamine sensitizers **SAS-1-5** with **N-719**

The photovoltaic performance of DSSCs cocktail co-sensitized with triphenylamine **SAS** sensitizers (**SAS-1-5**) and **N-719** was evaluated, and the key factors, including open-circuit voltage (*V*_*OC*_), short-circuit current (*J*_*SC*_ ​), fill factor (*FF*), and efficiency (*PCE*), are introduced in Table 5 and displayed in Fig. 8. These results shed light on the role of electron-acceptor strength in determining the efficiency of the sensitizers [50]. Dye loading experiments are commonly used to better realize the effect of several anchors on dye performance. Considering this, a DMF/H_2_O (1:1) combination containing 0.1 M NaOH was used to desorb dye from the TiO_2_ surface in order to quantify the total quantity of dye adsorbed on the TiO_2_.

**Table 5:** Photovoltaic factors of co-sensitizers **SAS-1-5** with **N-719**

Co-Sensitizer	V_OC_^a^ (V_OC_^b^)/eV	J_SC_^a^ (J_SC_^b^) (mA.cm^− 2^)	FF^a^ (FF^b^)/%	PCE^a^ (PCE^b^)/%	Concentration of the dye/10 ^− 5^ mol cm^− 2^
*N*-719	0.66 (0.65 ± 0.02)	20.40 (20.80 ± 0.73)	0.54 (0.55 ± 0.02)	7.33 (7.35 ± 0.02)	2.23
SAS-1 + *N*-719	0.67 (0.66 ± 0.02)	22.45 (22.48 ± 1.16)	0.55 (0.55 ± 0.01)	8.91 (8.84 ± 0.09)	2.45
SAS-2 + N-719	0.69 (0.66 ± 0.04)	24.27 (24.37 ± 2.85)	0.56 (0.57 ± 0.02)	9.12 (9.13 ± 0.19)	2.67
SAS-3 + N-719	0.64 (0.63 ± 0.02)	21.60 (20.98 ± 0.09)	0.53 (0.52 ± 0.03)	7.39 (7.33 ± 0.11)	2.29
SAS-4 + N-719	0.68 (0.656 ± 0.04)	23.80 (23.88 ± 2.20)	0.55 (0.54 ± 0.02)	9.02 (9.00 ± 0.27)	2.56
SAS-5 + N-719	0.68 (0.67 ± 0.01)	23.19 (22.98 ± 1.15)	0.56 (0.56 ± 0.01)	8.97 (8.90 ± 0.17)	2.39

The photovoltaic performance of DSSCs cocktail co-sensitized with **SAS** sensitizers (**SAS-1** to **SAS-5**) and **N-719** shows a clear correlation between the efficiency and the electron-withdrawing strength of the acceptor groups in the **SAS** dyes [51]. Among the cocktail co-sensitizer, (**SAS-2 + N-719)** exhibited the highest efficiency (9.12%) with a *V*_*OC*_ of 0.69 V and a *J*_*SC*_ of 24.27 mA/cm^2^, which can be attributed to the carboxylic cyanoacetamide acceptor group in **SAS-2**. This group provides a strong electron-withdrawing effect, which enhances electron injection efficiency, reduces recombination, and complements the spectral absorption of **N-719**. cocktail co-sensitizers (**SAS-4 + N-719**) and (**SAS-5 + N-719**), with efficiencies of 9.02% and 8.97%, respectively, followed closely due to their moderately strong acceptor groups that balance electron injection and dye regeneration. (**SAS-1 + N-719)** and (**SAS-3 + N-719**), with efficiencies of 8.91% and 7.39%, respectively, showed lower performance, reflecting the weaker electron-withdrawing capabilities of their acceptor groups, which may reduce the driving force for electron injection. The cocktail co-sensitizers of **N-719** and **SAS** dyes leverages complementary light absorption, increased dye loading, improved electron injection, reduced recombination, and synergistic electronic interactions to achieve higher photovoltaic performance.

**Fig. 9:**
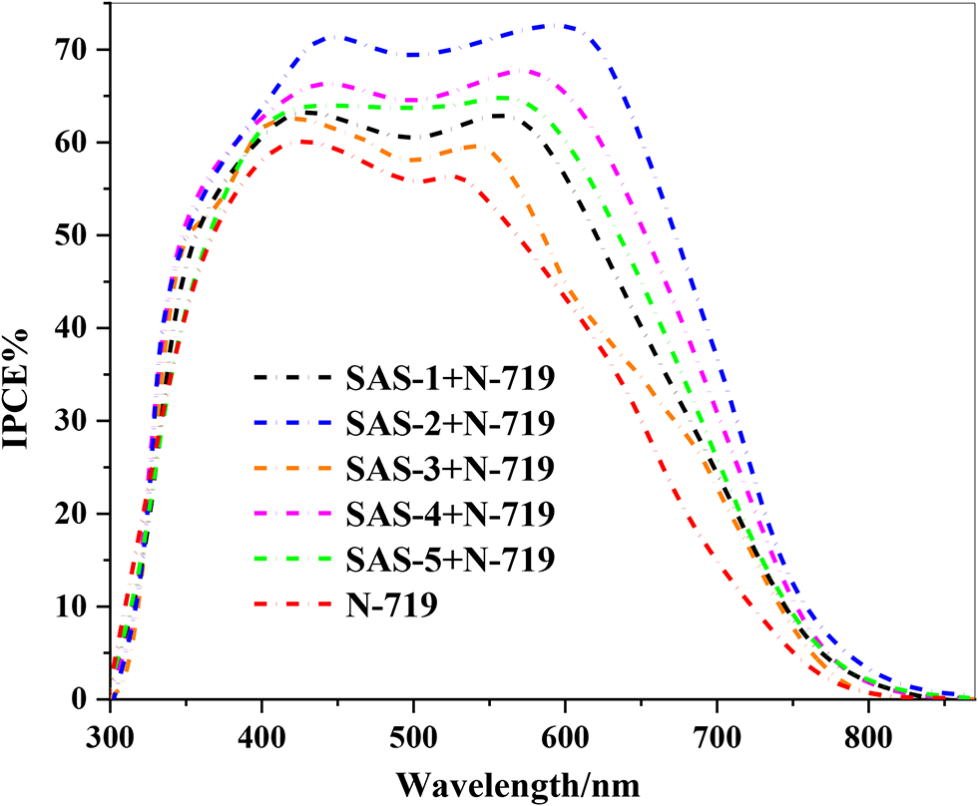
*IPCE* spectra of co-sensitized TPA-sensitizers **SAS-1-5** with **N-719**

The Incident-Photon-to-Current Efficiency (*IPCE)* spectra of **SAS-1** to **SAS-5** cocktail co-sensitized with **N-719** showed improved efficiency in converting light into electrical current across the visible spectrum (350–650 nm), demonstrating the benefits of combining the dyes [52]. Among the combinations, (**SAS-2 + N-719)** achieves the highest *IPCE*, reaching approximately 73% in the blue-green region (480–520 nm). This strong performance aligns with its high (*J*_*SC*_) and is attributed to the electron-withdrawing effect of **SAS-2’s** carboxylic cyanoacetamide acceptor, which works well with **N-719’s** strong absorption in the red region. Cocktail co-sensitizers (**SAS-4 + N-719**) and (**SAS-5 + N-719**) also perform well, with *IPCE* values around 67%, due to their ability to complement **N-719’s** absorption and enhance photon capture. In comparison, cocktail co-sensitizers (**SAS-1 + N-719**) and (**SAS-3 + N-719**) show lower *IPCE* values of about 58%, reflecting the weaker electron-accepting properties of their structures. cocktail co-sensitization with **N-719** expands the light absorption range and boosts efficiency by combining the strengths of both dyes [53]. Cocktail co-sensitizers **SAS-2** stands out as the most effective co-sensitizer, offering the best balance of light absorption and charge transfer (Fig. 9). As depicted in **(Fig.S29)**, the high photo-stability of the co-sensitizers **SAS-**dye for DSSC applications is confirmed by the fact that the cell exhibits excellent stability with no discernible degradation of the initial performances even after 300 h of illumination.

### Electrochemical Impedance Spectroscopy (EIS) for Sensitizers SAS-1-5

**Fig. 10:**
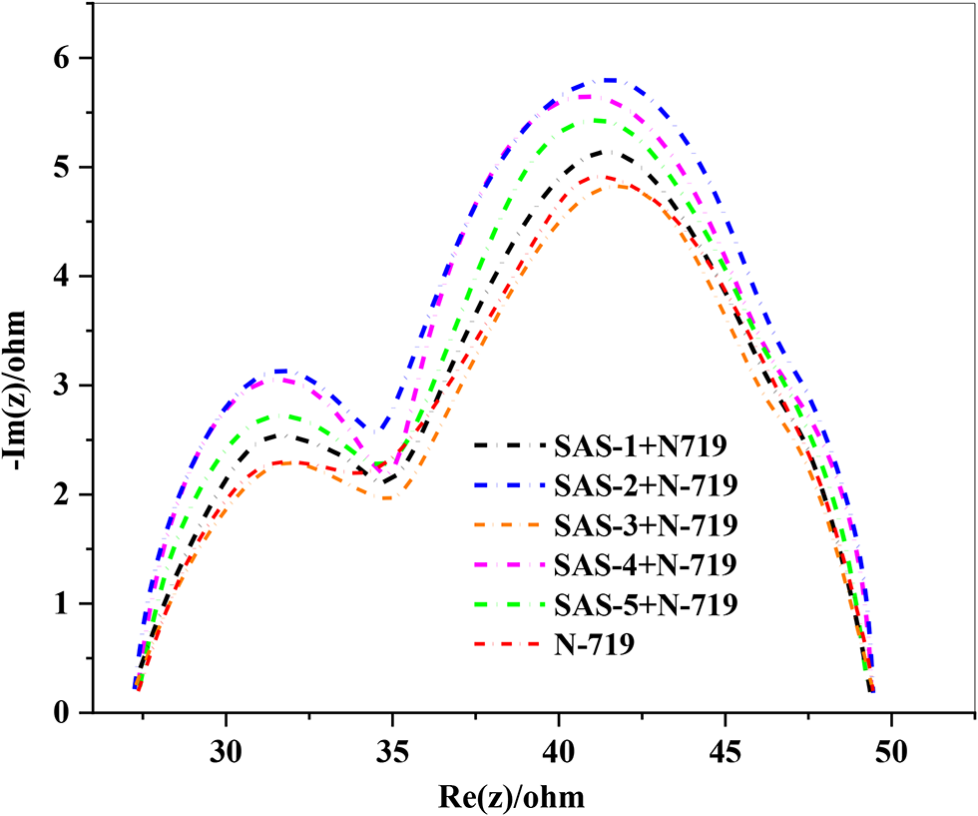
EIS Nyquist chart for sensitized and co-sensitized triphenylamine sensitizers **SAS-1-5** with **N-719**

EIS serves as a crucial analytical technique for evaluating interfacial electron recombination processes [54, 55] in (DSSCs) cocktail co-sensitized with SAS dyes (**SAS-1-5**) and **N-719**. As illustrated in Fig. 10, the Nyquist charts demonstrate two distinct semicircles in various frequency domains. The initial semicircle, observed at high frequencies, corresponds to the redox charge transfer resistance at the Pt/electrolyte interface (*Rpt*), while the subsequent semicircle at lower frequencies represents the charge transfer resistance at the TiO_2_/dye/electrolyte interface (*Rct*), which is closely associated with *V*_*OC*_. The Nyquist plots reveal that the order of *Rct* for the co-sensitized cells is (**SAS-2 + N-719**) > (**SAS-4 + N-719)** > (**SAS-5 + N-719**) > (**SAS-1 + N-719**) > **(N-719)** > (**SAS-3 + N-719**). These findings correlate well with the observed *V*_*OC*_ values reported in Table 4. The co-sensitized systems (**SAS-2 + N-719**, **SAS-4 + N-719**, and **SAS-5 + N-719**) exhibited higher *Rct* values compared to **N-719** alone, particularly for **SAS-2 + N-719**, which displayed the largest *Rct.* This enhancement can be attributed to the robust electron-withdrawing nature of the carboxylic cyanoacetamide anchoring group in **SAS-2**, which effectively reduces recombination processes at the TiO_2_/dye/electrolyte interface. Consequently, co-sensitization with (**SAS-2 + N-719**) significantly suppresses dark current, as reflected in its improved *V*_*OC*_. Similarly, the moderate Rct values observed for (**SAS-4 + N-719**) and (**SAS-5 + N-719**) indicate efficient recombination suppression and charge transfer dynamics. In contrast, co-sensitizers (**SAS-1 + N-719**) and (**SAS-3 + N-719**) exhibited lower *Rct*, consistent with their weaker anchoring groups and higher recombination rates. These findings confirm that cocktail co-sensitization, particularly with (**SAS-2 + N-719**), improves charge transfer resistance and reduces recombination, leading to enhanced photovoltaic performance in DSSCs.

## Conclusions

In conclusion, we successfully designed a diverse series of metal-free D-*π*-A dyes (**SAS-1** to **SAS-5**), incorporating a triphenylamine unit as the electron donor and cyanoacetamide (**SAS-1** and **SAS-2**), 2-(phenylsulfonyl)acetonitrile (**SAS-3**), and thiazolidine (**SAS-4** and **SAS-5**) as electron acceptors. These dyes were utilized as co-sensitizers in dye-sensitized solar cells (DSSCs). Molecular modeling revealed distinct charge separation in the HOMO and LUMO energy levels of all synthesized chromophores. In terms of optical properties, the **SAS-1** to **SAS-5** dyes displayed higher molar extinction coefficients in the UV-Vis region. Photovoltaic parameters were analyzed to elucidate the influence of various acceptor moieties on the overall device performance. The power conversion efficiencies (*PCEs*) of **SAS-1** to **SAS-5** co-sensitizers paired with **N-719** ranged from 7.39 to 9.12%. The absorption spectra of the co-sensitizers adsorbed onto mesoporous TiO₂ aligned well with the observed *PCE* values. Notably, **SAS-2**, containing anchoring groups (COOH, CN, and NH), achieved the highest photoconversion efficiency (9.12%) with the maximum open-circuit voltage (*V*_*OC*_) of 0.69 V. This performance was attributed to its superior dye loading capacity, as well as the highest short-circuit current density (*J*_*SC*_, 11.84 mA/cm²) and incident photon-to-current efficiency (*IPCE*, 70%), driven by its exceptional molar extinction coefficient for (ICT). These findings demonstrate that the integration of **SAS-1** to **SAS-5** with **N-719** offers a promising strategy for enhancing DSSC efficiency.

## Electronic Supplementary Material

Below is the link to the electronic supplementary material.

Supplementary Material 1

## Data Availability

No datasets were generated or analysed during the current study.

## Declarations

### Dual Publication

The results/data/figures in this manuscript have not been published elsewhere, nor are they under consideration (from you or one of your Contributing Authors) by another publisher.

### Third Party Material

All the material is owned by the authors and/or no permissions are required.
