# Supplementary material for: Triphenylamine-Based Metal-Free Organic Dyes as Co-Sensitizers: Enhancing Dye-Sensitized Solar Cell Performance Through Innovative Molecular Design
Source: J Fluoresc. 2025 May 16;35(11):10925–38. doi: 10.1007/s10895-025-04340-9 (PMC12718282; doi:10.1007/s10895-025-04340-9)
Supplement: Supplementary file 1 — Supplementary Material 1 [file 10895_2025_4340_MOESM1_ESM.docx]

**Triphenylamine-Based Metal-Free Organic Dyes as Co-Sensitizers: Enhancing Dye-Sensitized Solar Cell Performance through Innovative Molecular Design**

Samar E. Mahmoud^1^, Safa A. Badawy*^1^, Ahmed A. Fadda^1^, Ehab Abdel-Latif^1^, Mohamed R. Elmorsy^1,2^.

*^1^ Department of Chemistry, Faculty of Science, Mansoura University, El-Gomhoria Street, 35516 Mansoura, Egypt.*

*^🖂^Corresponding author: E-mail:* [safabadawy140@gmail.com](mailto:safabadawy140@gmail.com), [m.r.elmorsy@gmail.com](mailto:m.r.elmorsy@gmail.com)

1. **Experimental**
   1. ***Materials and equipments***

The chemicals and reagents used in the synthesis of target dyes such as triphenylamine, terephthalaldehyde, triphenylphosphine hydrobromide, sodium borohydride, and 18-crown-6 were obtained from Sigma Aldrich and Alfa Aesar. Silica gel (60-120 mm) was used for column chromatography. The IR spectra were determined by using A Nicolet iS10 spectrometer. NMR spectra (^1^H and ^13^C NMR) of synthesized dyes were measured in DMSO-*d*_6_ at 500 MHz and 125 MHz respectively and were recorded by JEOL’s NMR spectrometer. Mass spectra were taken on through Thermo Scientific GC/MS model ISQ. UV-Vis absorption spectra of all sensitizers were estimated at room temperature using the high-performance double-beam spectrophotometer (T80 series). The elemental analysis of C, H, and N was obtained using the Perkin-Elmer 2400 analyzer. Finally, all DSSCs fabrications and instruments were thoroughly explained.

- 1. ***Synthesis***
     1. ***Synthesis of 4-(4-(diphenylamino)styryl)benzaldehyde (5):***

In a three-necked flask, the mixture of terephthalaldehyde **4** (0.26 g, 2 mmol), 18-crown-6 (0.03 g, 0.10 mmol), and K_2_CO_3_ (0.55 g, 4 mmol) was added, then stirred for 30 min. the solution of phosphonium salt **3** (1.20 g, 2 mmol) in dry DMF (20 mL) was added dropwise to the mixture over 3 h at 70°C under nitrogen. The reaction mixture was stirred overnight at 70°C, then poured into mashed ice. The formed solid was purified by recrystallization with ethanol and drops of acetone to give yellow aldehyde **5**.

Yield (98%); m.p: 130-132°C. IR (cm^-1^): 1690 (C=O), 1585 (C=C). ^1^H NMR: 6.94 (d, *J* = 9.00 Hz, 2H, Ar-H), 7.03-7.09 (m, 6H, Ar-H), 7.19 (d, *J* = 16.00 Hz, 1H, CH=C), 7.32 (t, *J* = 8.00 Hz, 4H, Ar-H), 7.41 (d, *J* = 16.00 Hz, 1H, CH=C), 7.54 (d, *J* = 9.00 Hz, 2H, Ar- H), 7.76 (d, *J* = 8.00 Hz, 2H, Ar-H), 7.87 (d, *J* = 8.00 Hz, 2H, Ar-H), 9.95 (s, 1H, CHO). ^13^C NMR: 122.38 (2C), 123.57 (2C), 124.46 (4C), 125.42, 126.42, 126.69 (2C), 128.16 (2C), 129.65 (4C), 130.03 (2C), 130.41, 131.53, 134.72, 143.50, 146.77, 147.47, 192.29. Analysis for C_27_H_21_NO (375.16): Calculated: C, 86.37; H, 5.64; N, 3.73%. Found: C, 86.53; H, 5.70; N, 3.71%.

- - 1. ***General method for synthesis of 5-(5-oxo-3-phenylthiazolidin-2-ylidene)pyrimidine-2,4,6(1H,3H,5H)-trione (7e):***

In a 100 mL conical flask, the barbituric acid **6** (1.02 g, 8 mmol) was stirred with phenyl isothiocyanate (0.96 mL, 8 mmol) and KOH (0.45 g, 8 mmol) in dry *N*,*N*-dimethylformamide (20 mL) for 6 h. Then, chloroacetyl chloride (0.64 mL, 8 mmol) was added drop by drop to the solution and continued stirring overnight. The mixture was poured into crushed ice and neutralized with diluted HCl. Finally, the solid formed was filtrated, dried, and recrystallized by heating in acetic acid to obtain pale orange powder.

Yield (89%); m.p. > 300°C. R (cm^-1^): 3464 and 3392 (N-H), 2969 and 2928 (C-H, aliphatic), 1708, 1644 (C=O) and 1570 (C=C). ^1^H NMR: 4.17 (s, 2H, CH_2_), 7.32 (t, *J* = 7.00 Hz, 3H, Ar-H), 7.44 (d, *J* = 8.00 Hz, 2H, Ar-H), 11.65 (br. s, 1H, N-H), 12.38 (br. s, 1H, N-H). Analysis for C_13_H_9_N_3_O_4_S (303.03): Calculated: C, 51.48; H, 2.99; N, 13.85%. Found: C, 51.59; H, 2.98; N, 13.80%.

- - 1. ***Synthesis of 2-cyano-3-(4-(4-(diphenylamino)styryl)phenyl)-N-(4-nitrophenyl)acrylamide (SAS-1):***

A mixture of aldehyde **5** (0.37 g, 1 mmol), 2-cyano-*N*-(4-(nitro)phenyl)acetamide (**7a**) (0.21 g, 1 mmol), NH_4_OAc (0.20 g), and glacial AcOH (25 mL) was refluxed for 2 h. The pure sensitizer that formed on hot was filtrated after cooling to yield **SAS-1** as a deep red powder.

Yield (56%); m.p. > 300°C. IR (cm^-1^): 3325 (N-H), 2218 (C≡N), 1689 (C=O), 1570 (C=C). ^1^H NMR: 6.94 (d, *J* = 9.00 Hz, 2H, Ar-H), 7.04-7.09 (m, 6H, Ar-H), 7.19 (d, *J* = 16.00 Hz, 1H, CH=C), 7.32 (t, *J* = 8.00 Hz, 4H, Ar-H), 7.41 (d, *J* =16.00 Hz, 1H, CH=C), 7.54 (d, *J* = 9.00 Hz, 2H, Ar- H), 7.78 (d, *J* = 8.50 Hz, 2H, Ar-H), 7.94 (d, *J* = 9.00 Hz, 2H, Ar-H), 8.01 (d, *J* = 9.00 Hz, 2H, Ar-H), 8.27 (s, 1H, CH=C), 8.29 (d, *J* = 8.50 Hz, 2H, Ar-H), 10.92 (s, 1H, N-H). ^13^C NMR: 105.22, 116.43, 120.26 (2C), 122.41 (2C), 123.72 (2C), 124.59 (4C), 124.98 (2C), 125.45, 126.99 (2C), 128.28 (2C), 129.78 (4C), 13.24, 130.47, 131.09 (2C), 131.53, 142.20, 142.97, 144.68, 146. 82 (2C), 147.58, 151.28, 161.66. Mass analysis (m/z, %): 562 (M^+^, 26.38), 553 (77.37), 536 (56.25), 512 (46.06), 491 (52.97), 476 (77.13), 459 (51.95), 401 (89.66), 390 (67.61), 380 (66.92), 329 (100.00), 313 (83.35), 287 (38.64), 173 (71.03), 153 (80.35), 102 (91.08). Analysis for C_36_H_26_N_4_O_3_ (562.20): Calculated: C, 76.85; H, 4.66; N, 9.96%. Found: C, 76.95; H, 4.60; N, 9.87%.

- - 1. ***4-(2-Cyano-3-(4-(4-(diphenylamino)styryl)phenyl)acrylamido)benzoic acid (SAS-2):***

Ammonium acetate (0.10 g) was added to a suspension of compound **5** (0.37 g, 1 mmol) and 4-(2-cyanoacetamido)benzoic acid (**7b**) (0.20 g, 1 mmol) in acetic acid (20 mL). After seven hours of boiling, the mixture was allowed to cool to room temperature (37°C). The collected precipitate was filtered and washed with ethanol to give **SAS-2** as a dark orange dye.

Yield (49%); m.p: 230-232°C. IR (cm^-1^): 3328 (N-H), 2548 (COOH), 2221 (C≡N), 1694 (C=O), 1581 (C=C). ^1^H NMR: 6.94 (d, *J* = 8.50 Hz, 2H, Ar-H), 7.03-7.09 (m, 6H, Ar-H), 7.19 (d, *J* = 16.00 Hz, 1H, CH=C), 7.32 (t, *J* = 8.00 Hz, 4H, Ar-H), 7.41 (d, *J* = 16.00 Hz, 1H, CH=C), 7.54 (d, *J* = 8.50 Hz, 2H, Ar- H), 7.76-7.81 (m, 4H, Ar-H), 7.94 (d, *J* = 9.00 Hz, 2H, Ar-H), 8.00 (d, *J* = 8.00 Hz, 2H, Ar-H), 8.25 (s, 1H, CH=C), 10.65 (s, 1H, N-H), 12.44 (brs, 1H, COOH). ^13^C NMR: 105.49, 116.48, 119.76 (2C), 122.40 (2C), 123.62 (2C), 124.51 (4C), 125.44, 126.15, 126.90 (2C), 128.18 (2C), 129.69 (4C), 130.33 (2C), 130.47, 130.91 (2C), 131.34, 142.40, 142.43, 146.79 (2C), 147.50, 150.66, 161.19, 166.89, 172.07. Mass analysis (m/z, %): 561 (M^+^, 48.27), 552 (26.20), 473 (76.98), 472 (63.86), 446 (40.04), 442 (48.84), 326 (66.99), 206 (51.83), 179 (63.10), 167 (100.00), 145 (54.33), 131 (49.29), 97 (40.80), 50 (39.01). Analysis for C_37_H_27_N_3_O_3_ (561.21): Calculated: C, 79.13; H, 4.85; N, 7.48%. Found: C, 79.27; H, 4.82; N, 7.43%.

- - 1. ***3-(4-(4-(Diphenylamino)styryl)phenyl)-2-(phenylsulfonyl)acrylonitrile (SAS-3):***

A dry RB flask (50 mL) was filled with a combination of 4-(4-(diphenylamino)styryl)benzaldehyde (**5**) (0.37 g, 1 mmol), 2-(phenylsulfonyl)acetonitrile (**7c**) (0.54 g, 1 mmol), and NH_4_OAc (0.22 g). After dissolving this combination in 25 mL of glacial acetic acid, it was refluxed for 14 h. The reaction mass was left to cool at 25°C once the reaction was finished. The targeting solid was filtered and dried. The resulting crude was purified by column chromatography using silica gel as stationary phase and petroleum ether:ethyl acetate (9:1) as the mobile phase to get dark red powder.

Yield (51%); m.p: 160-162°C. IR (cm^-1^): 2213 (C≡N), 1577 (C=C), 1327 and 1314 (SO_2_). ^1^H NMR: 6.93 (d, *J* = 8.50 Hz, 2H, Ar-H), 7.03-7.09 (m, 6H, Ar-H), 7.19 (d, *J* = 16.00 Hz, 1H, CH=C), 7.32 (t, *J* = 8.00 Hz, 4H, Ar-H), 7.46 (d, *J* = 16.00 Hz, 1H, CH=C), 7.53 (d, *J* = 9.00 Hz, 2H, Ar- H), 7.72-7.77 (m, 4H, Ar-H), 7.83 (t, *J* = 8.00 Hz, 1H, Ar-H), 8.02 (t, *J* = 8.00 Hz, 4H, Ar-H), 8.49 (s, 1H, CH=C). ^13^C NMR: 111.31, 113.69, 122.16 (2C), 123.74 (2C), 124.63 (4C), 125.13, 126.98 (2C), 128.06 (2C), 128.36 (2C), 128.54 (2C), 129.73 (4C), 130.20 (2C), 131.85 (2C), 132.44, 135.07, 137.91, 143.78, 146.71 (2C), 147.72, 152.60. Mass analysis (m/z, %): 538 (M^+^, 60.33), 510 (32.61), 478 (35.06), 455 (28.30), 418 (29.91), 389 (56.35), 343 (30.32), 341 (28.81), 327 (31.05), 301 (33.13), 289 (33.64), 287 (51.11), 222 (67.97), 216 (60.20), 196 (100.00), 188 (38.88). Analysis for C_35_H_26_N_2_O_2_S (538.17): Calculated: C, 78.04; H, 4.87; N, 5.20%. Found: C, 78.16; H, 4.80; N, 5.23%.

- - 1. ***2-(4-(4-(4-(Diphenylamino)styryl)benzylidene)-5-oxo-3-phenylthiazolidin-2-ylidene)malononitrile (SAS-4):***

A mixture of 4-(4-(diphenylamino)styryl)benzaldehyde (**5**) (0.37 g, 1 mmol) and thiazolidine-5-one derivative (**7d**) (0.24 g, 1 mmol) in glacial acetic acid (15 mL) and ammonium acetate (0.20 g) was refluxed for 3 h. The precipitate obtained on hot was washed with H_2_O and purified by recrystallization with boiling ethanol to obtain a red powder.

Yield (53%); m.p: 280-282°C. IR (cm^-1^): 2216 (C≡N), 1714 (C=O), 1574 (C=C). ^1^H NMR: 6.94 (d, *J* = 9.00 Hz, 2H, Ar-H), 7.04-7.09 (m, 6H, Ar-H), 7.19 (d, *J* = 16.00 Hz, 1H, CH=C), 7.32 (t, *J* = 8.00 Hz, 4H, Ar-H), 7.38 (d, *J* = 16.00 Hz, 1H, CH=C), 7.54-7.60 (m, 7H, Ar- H), 7.73 (d, *J* = 8.50 Hz, 2H, Ar-H), 7.79 (d, *J* = 9.00 Hz, 2H, Ar-H), 8.04 (s, 1H, CH=C). ^13^C NMR: 109.88, 113.96, 116.56, 122.43 (2C), 123.59 (2C), 124.46 (4C), 125.47, 127.31 (2C), 128.14 (2C), 129.27 (2C), 129.59 (4C), 129.66 (4C), 129.98, 130.52, 131.03, 131.21 (2C), 133.20, 135.00, 140.64, 146.77 (2C), 147.43, 165.86, 167.02. Mass analysis (m/z, %): 598 (M^+^, 21.91), 582 (51.83), 564 (32.89), 533 (62.38), 526 (47.43), 503 (72.16), 498 (49.50), 395 (37.43), 365 (58.24), 362 (50.68), 360 (45.25), 264 (49.13), 261 (37.83), 136 (46.57), 110 (64.08), 77 (59.29), 56 (100.00). Analysis for C_39_H_26_N_4_OS (598.18): Calculated: C, 78.24; H, 4.38; N, 9.36%. Found: C, 78.13; H, 4.42; N, 9.43%.

- - 1. ***5-(4-(4-(4-(Diphenylamino)styryl)benzylidene)-5-oxo-3-phenylthiazolidin-2-ylidene)pyrimidine-2,4,6(1H,3H,5H)-trione (SAS-5):***

In 50 mL round bottom flask, the solution of the substituted benzaldehyde **5** (0.37 g, 1 mmol) was refluxed with 5-(5-oxo-3-phenylthiazolidin-2-ylidene)pyrimidine-2,4,6(1*H*,3*H*,5*H*)-trione (**7e**) (0.30 g, 1 mmol) and NH_4_OAc (0.5 g) in AcOH (25 mL) for 14 h. The solid formed by pouring it into cool water was collected, washed with ethanol, and dried. The residue was purified by using silica gel column chromatography using petroleum ether/ethyl acetate (2:1) as the mobile phase to produce dark red sensitizer. Yield (59%); m.p. > 300°C. IR (cm^-1^): 3246 and 3177 (N-H), 1695 and 1640 (C=O), 1590 (C=C). ^1^H NMR (ppm): 6.94 (d, *J* = 8.50 Hz, 2H, Ar-H), 7.02-7.05 (m, 9H, Ar-H), 7.30-7.33 (m, 7H, Ar-H), 7.43 (s, 1H, CH=C), 7.44 (d, *J* = 9.00 Hz, 4H, Ar-H), 7.66-7.72 (m, 3H, Ar- H). ^13^C NMR (ppm): 109.86, 116.56, 122.53 (2C), 123.58 (2C), 124.47 (2C), 125.59 (4C), 127.41 (2C), 128.14 (2C), 129.26 (2C), 129.58 (2C), 129.65 (4C), 130.51, 131.03, 131.20, 133.30, 135.10 (2C), 141.21, 145.11, 145.96, 146.70, 147.43, 150.02, 154.00, 164.86 (2C), 170.01. Mass analysis (m/z, %): 660 (M^+^, 32.39), 652 (46.48), 540 (29.80), 447 (50.31), 437 (73.28), 334 (44.89), 264 (100.00), 227 (89.70), 198 (83.70), 135 (51.52), 84 (64.57), 56 (64.57). Analysis for C_40_H_28_N_4_O_4_S (660.18): Calculated: C, 72.71; H, 4.27; N, 8.48%. Found: C, 72.83; H, 4.32; N, 8.57%.

1. **Analytical Measurements**

**Figure (S1): UV-Vis. absorption of N719**

| **λ_max_ (nm)** | **ε (10^4^M^-1^ cm^-1^)** | **λ_onset_ / nm** | ***E*_0-0_ (ev)** |
| --- | --- | --- | --- |
| **307, 384, 537** | **3.69, 1.18, 1.08** | **602** | **1.92** |

**
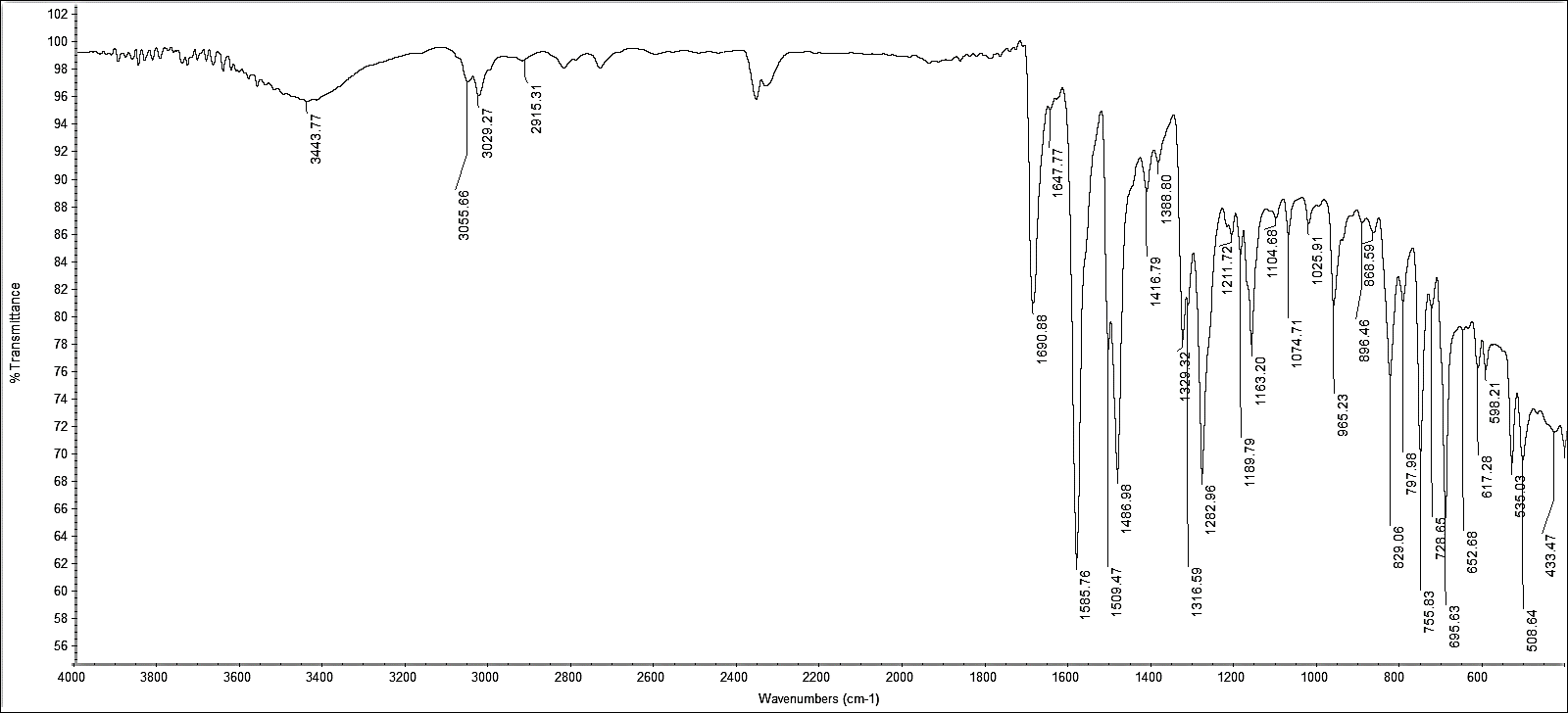
**

**Figure (S2): IR spectrum of compound 5.**


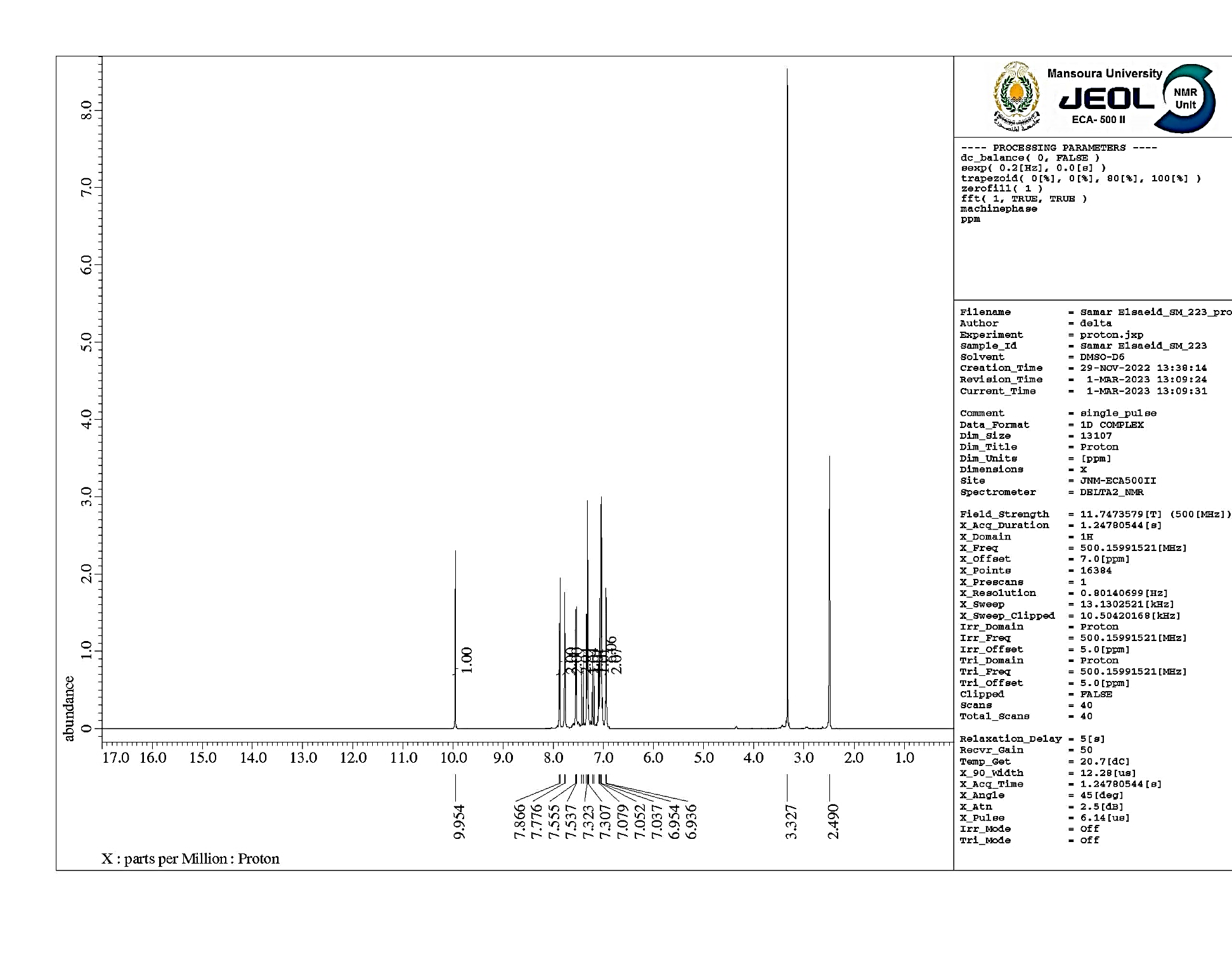

**Figure (S3): ^1^H NMR spectrum of compound 5.**


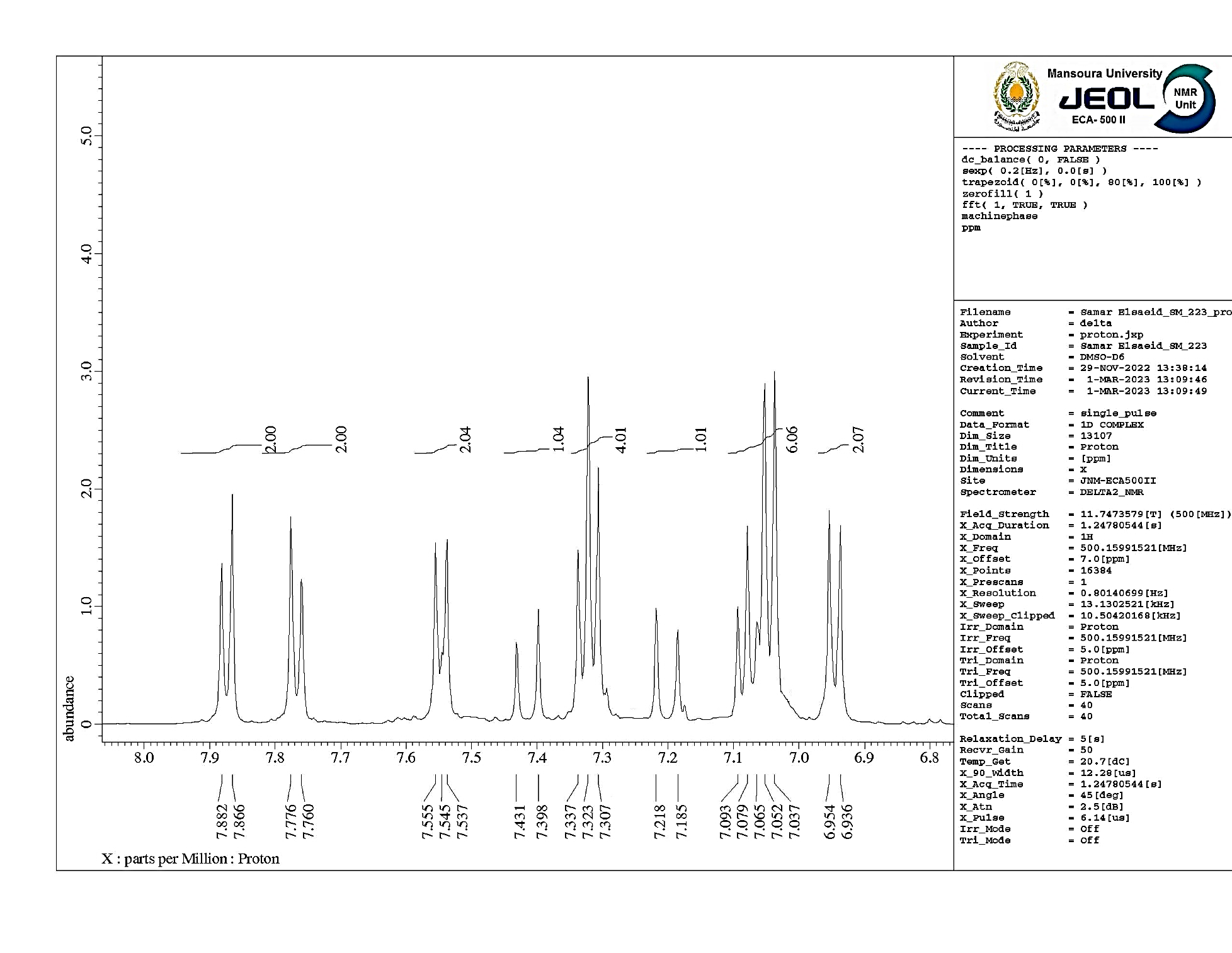

**Figure (S4): ^1^H NMR spectrum of compound 5.**


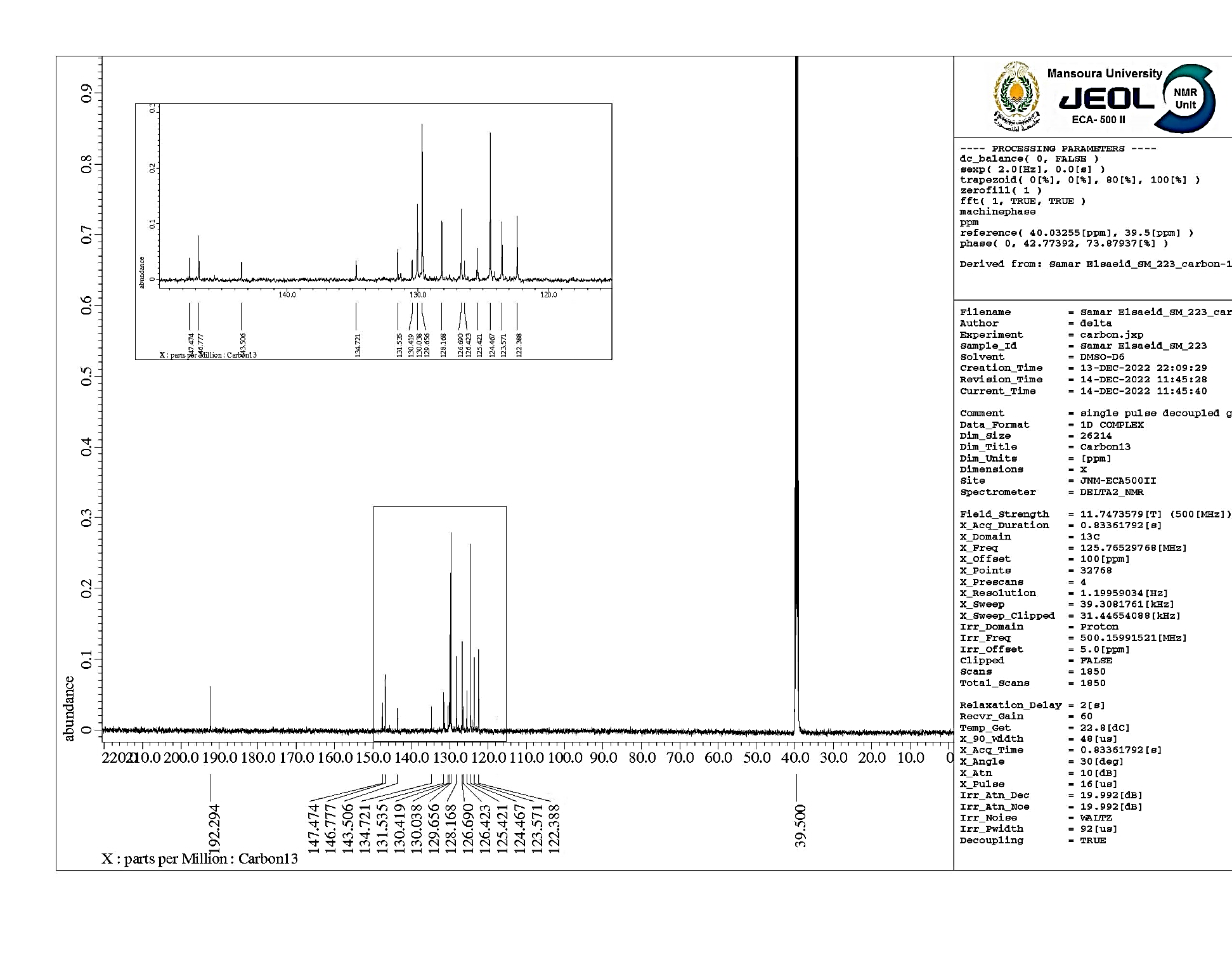

**Figure (S5): ^13^C NMR spectrum of compound 5.**

**
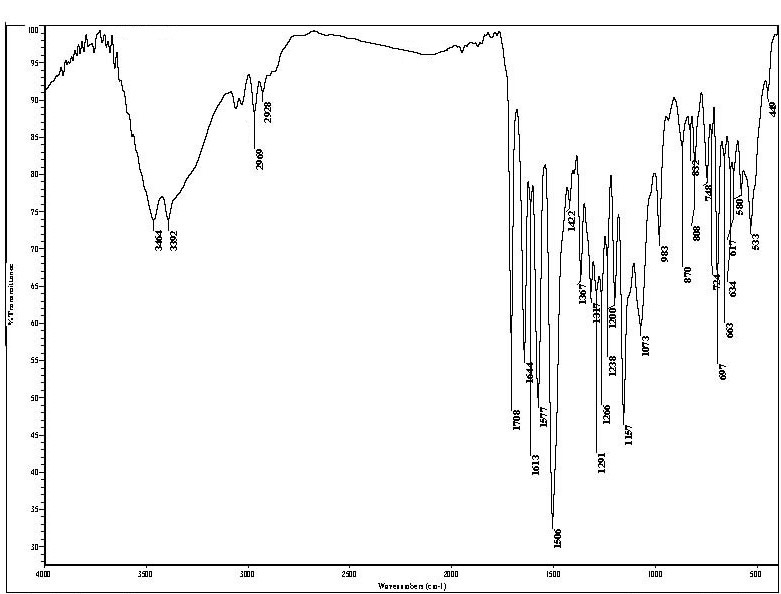
**

**Figure (S6): IR spectrum of compound 7e.**

**
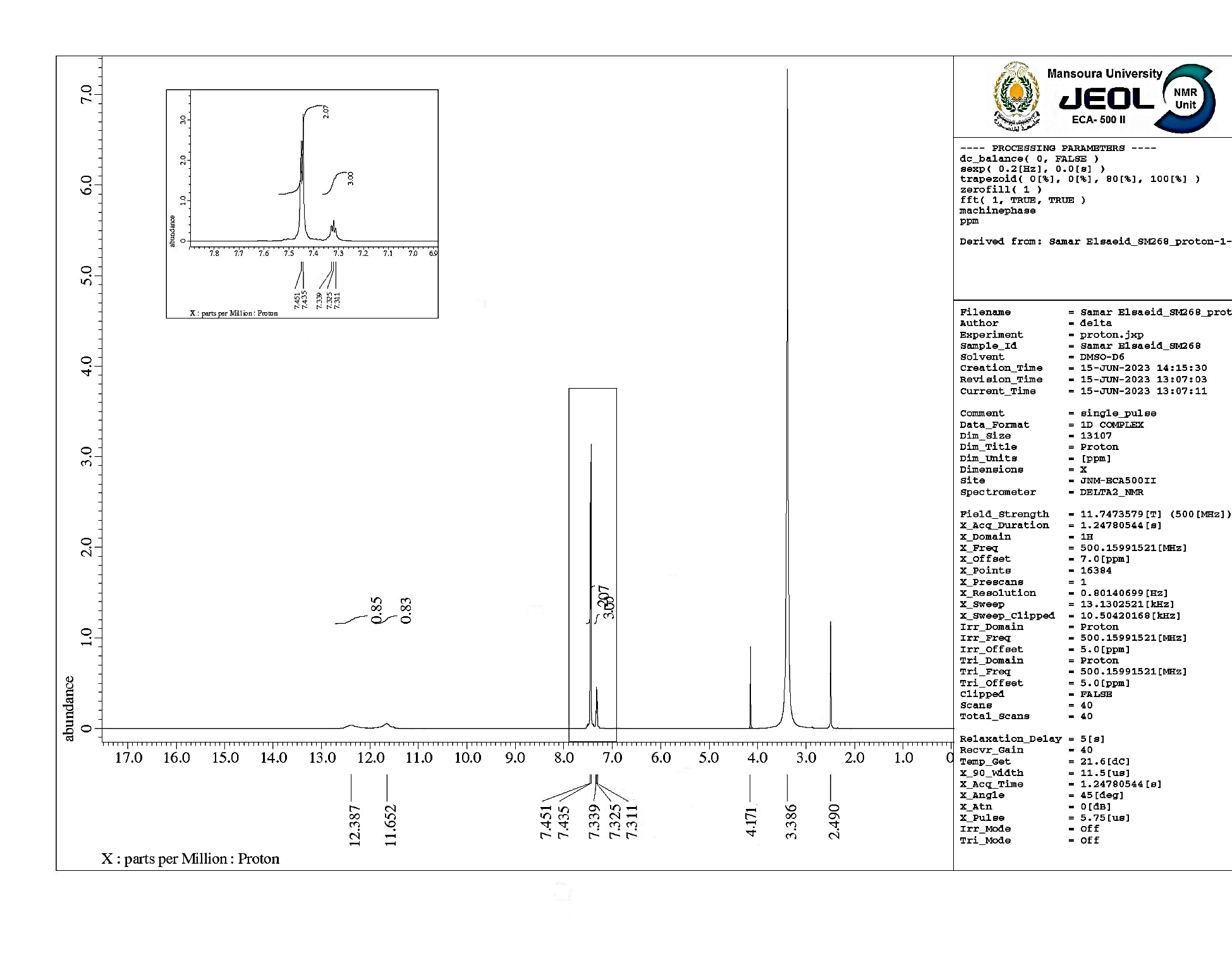
**

**Figure (S7): ^1^H NMR spectrum of compound 7e.**

**
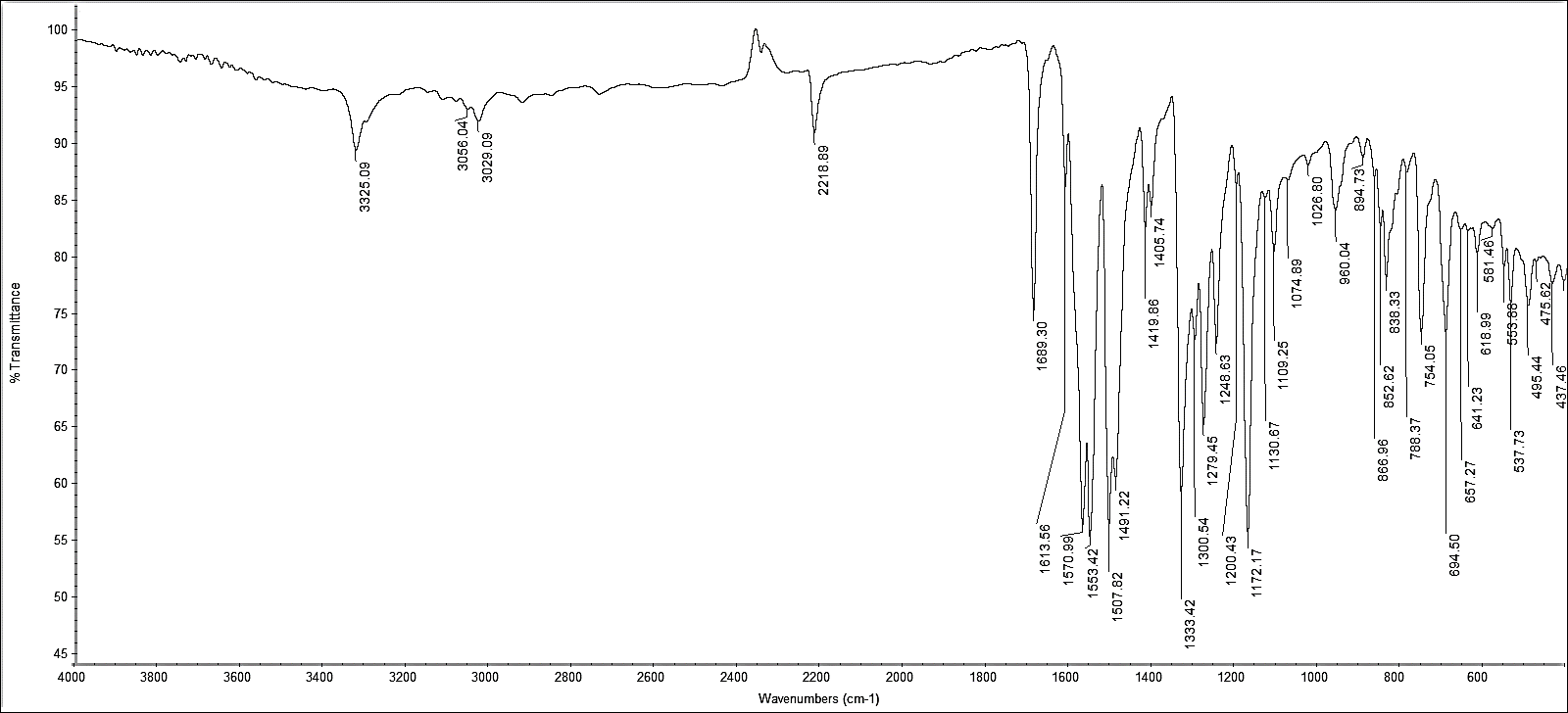
**

**Figure (S8): IR spectrum of dye SAS-1.**

**
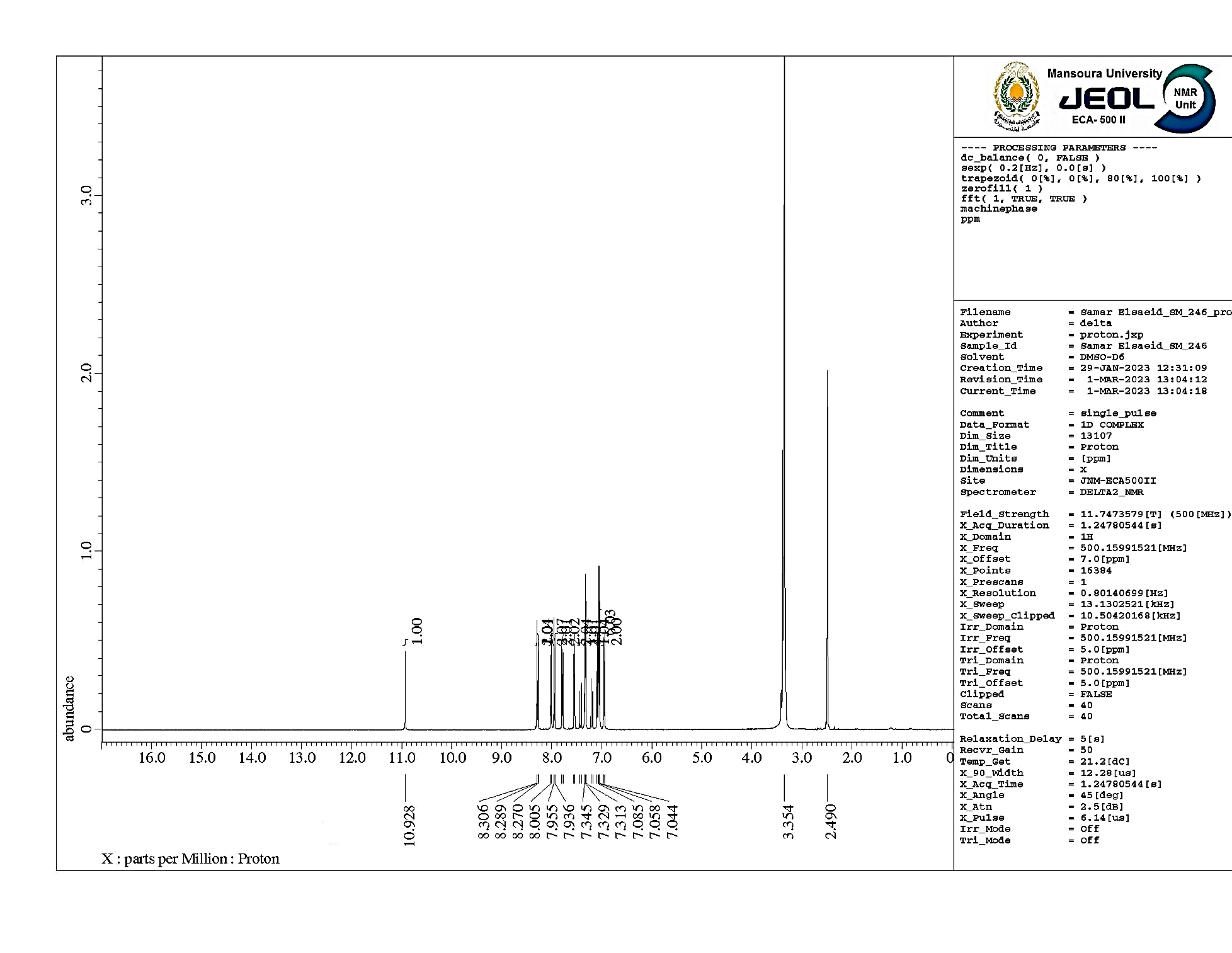
**

**Figure (S9): ^1^H NMR spectrum of dye SAS-1.**

**
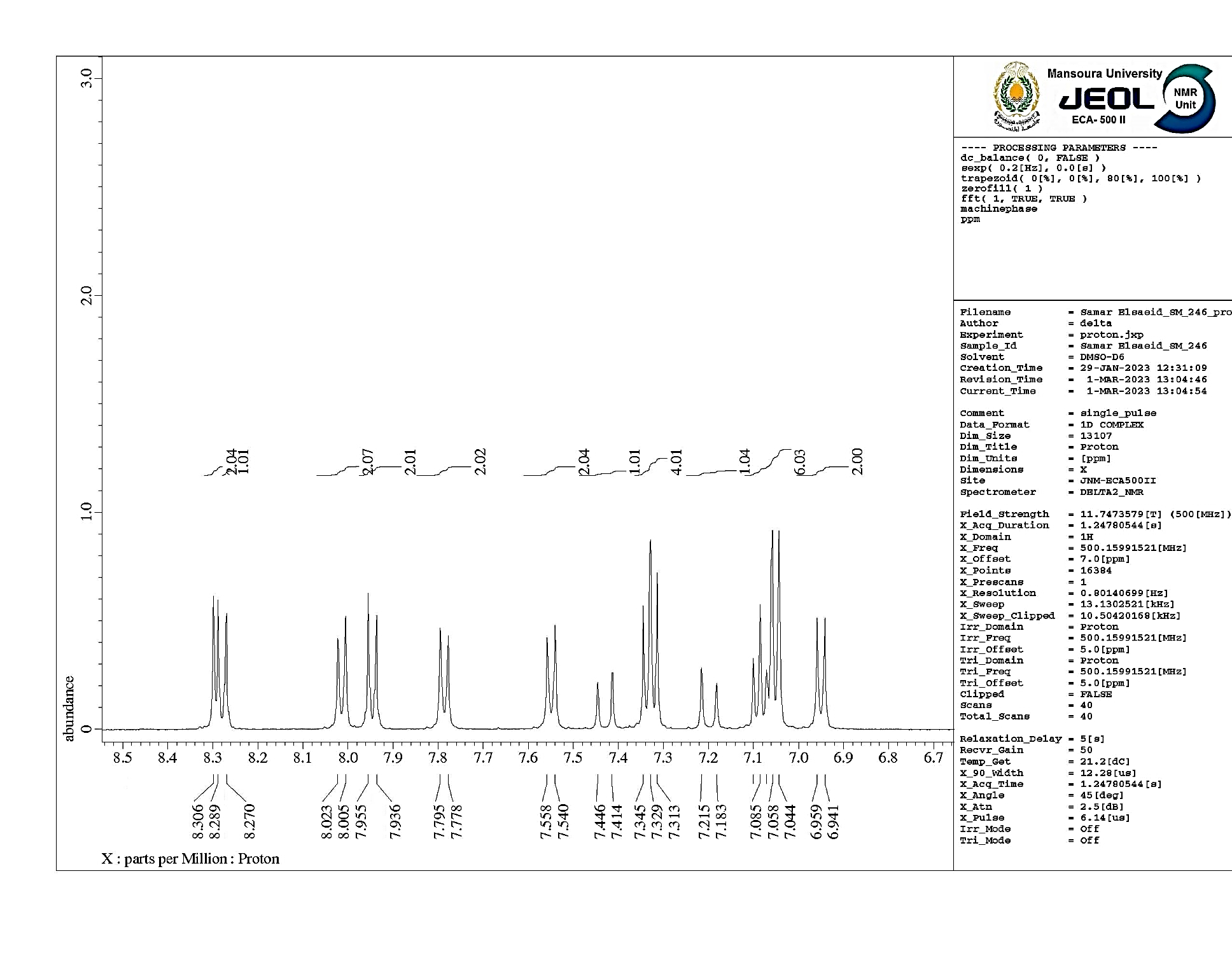
**

**Figure (S10): ^1^H NMR spectrum of dye SAS-1.**

**
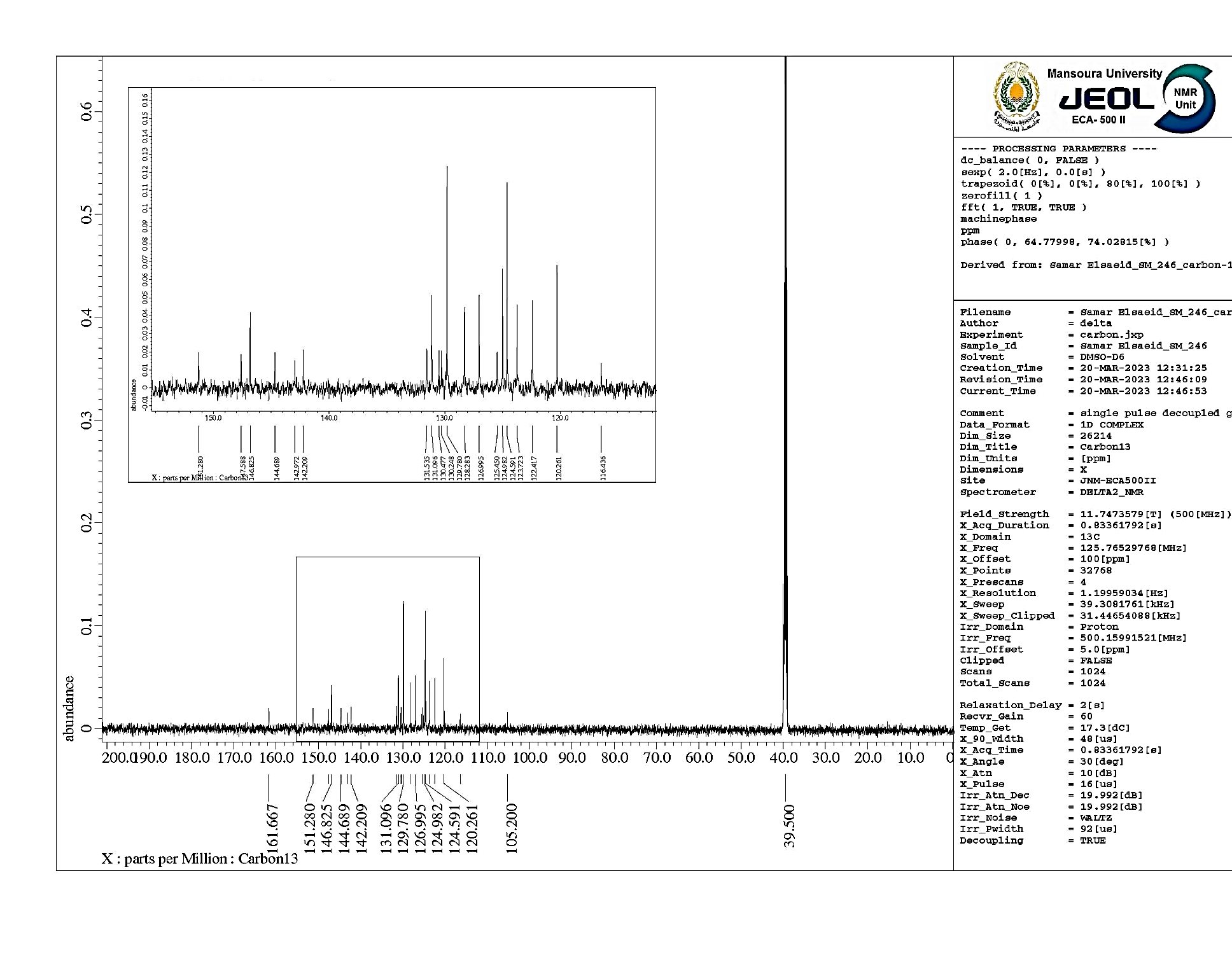
**

**Figure (S11): ^13^C NMR spectrum of dye SAS-1.**

**Figure (S12): Mass spectrum of dye SAS-1.**


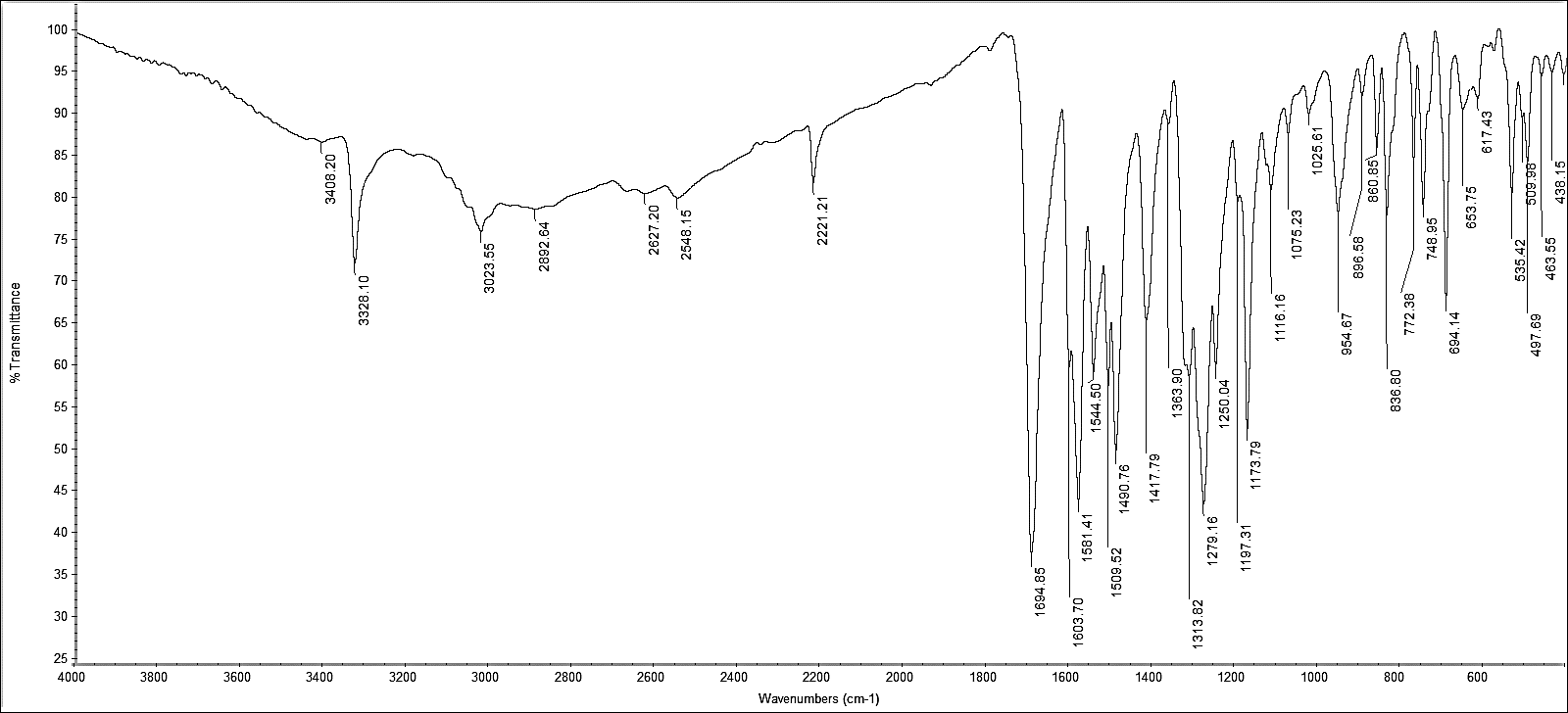

**Figure (S13): IR spectrum of dye SAS-2.**


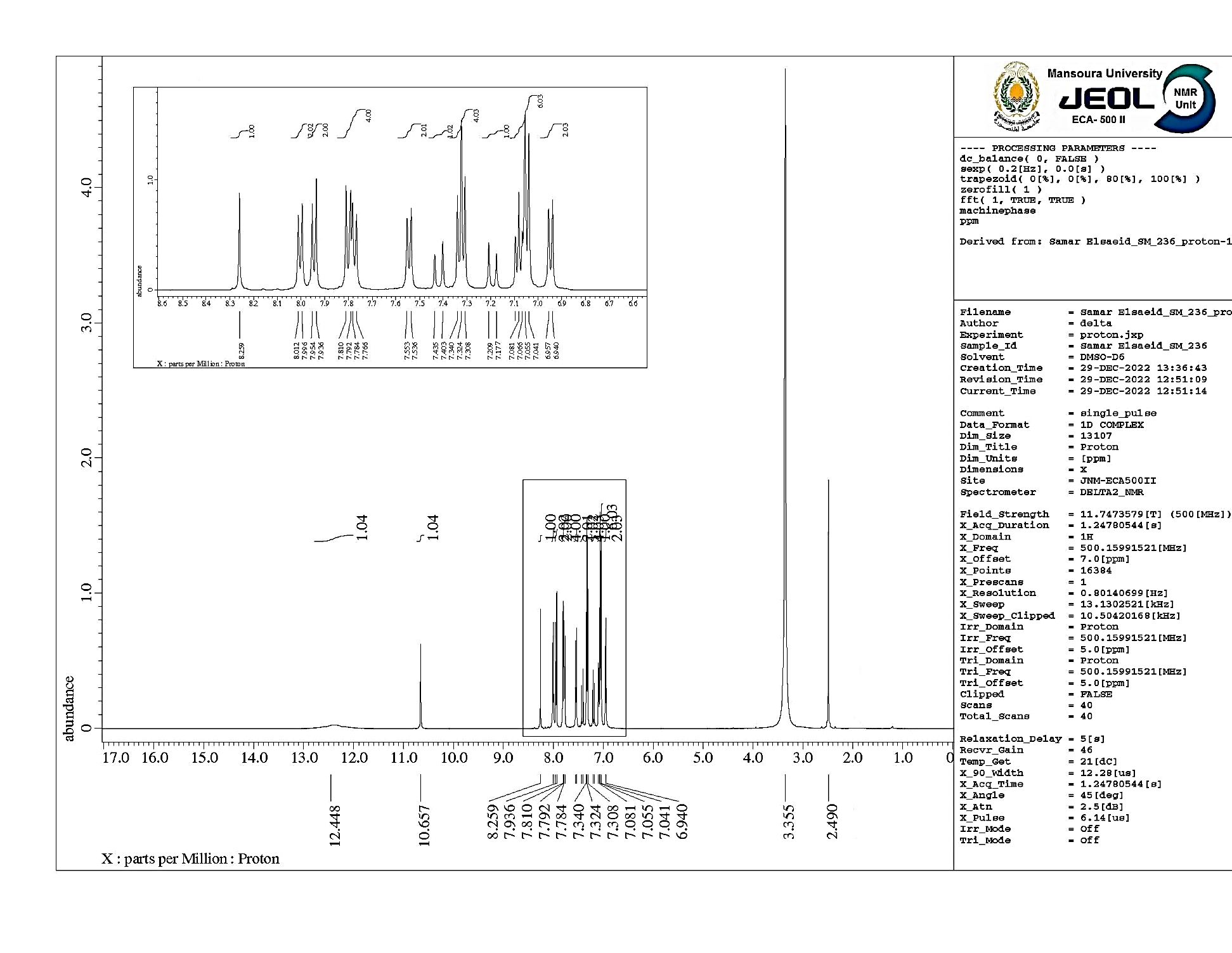

**Figure (S14): ^1^H NMR spectrum of dye SAS-2.**

**
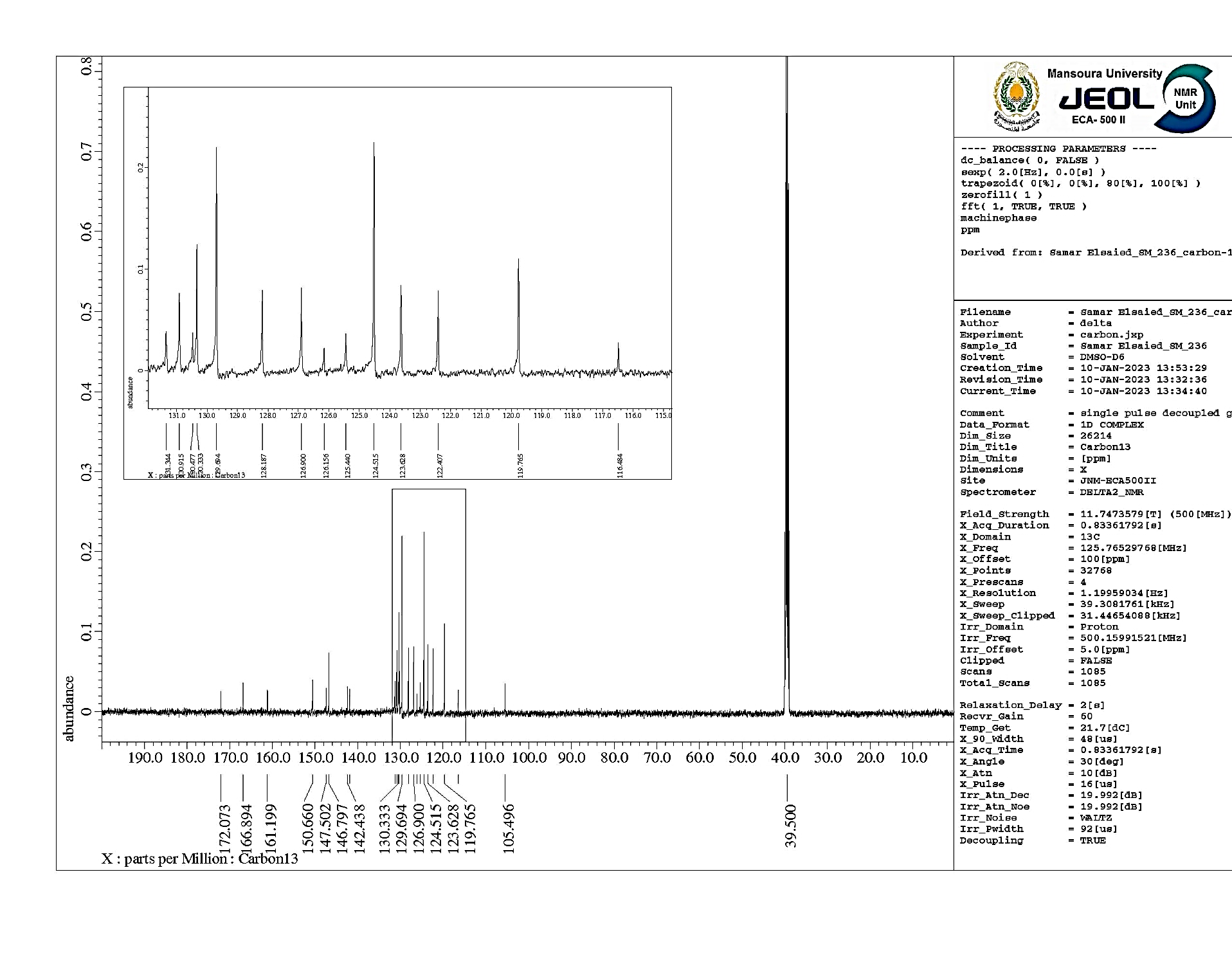
**

**Figure (S15): ^13^C NMR spectrum of dye SAS-2.**

**Figure (S16): Mass spectrum of dye SAS-2.**

**
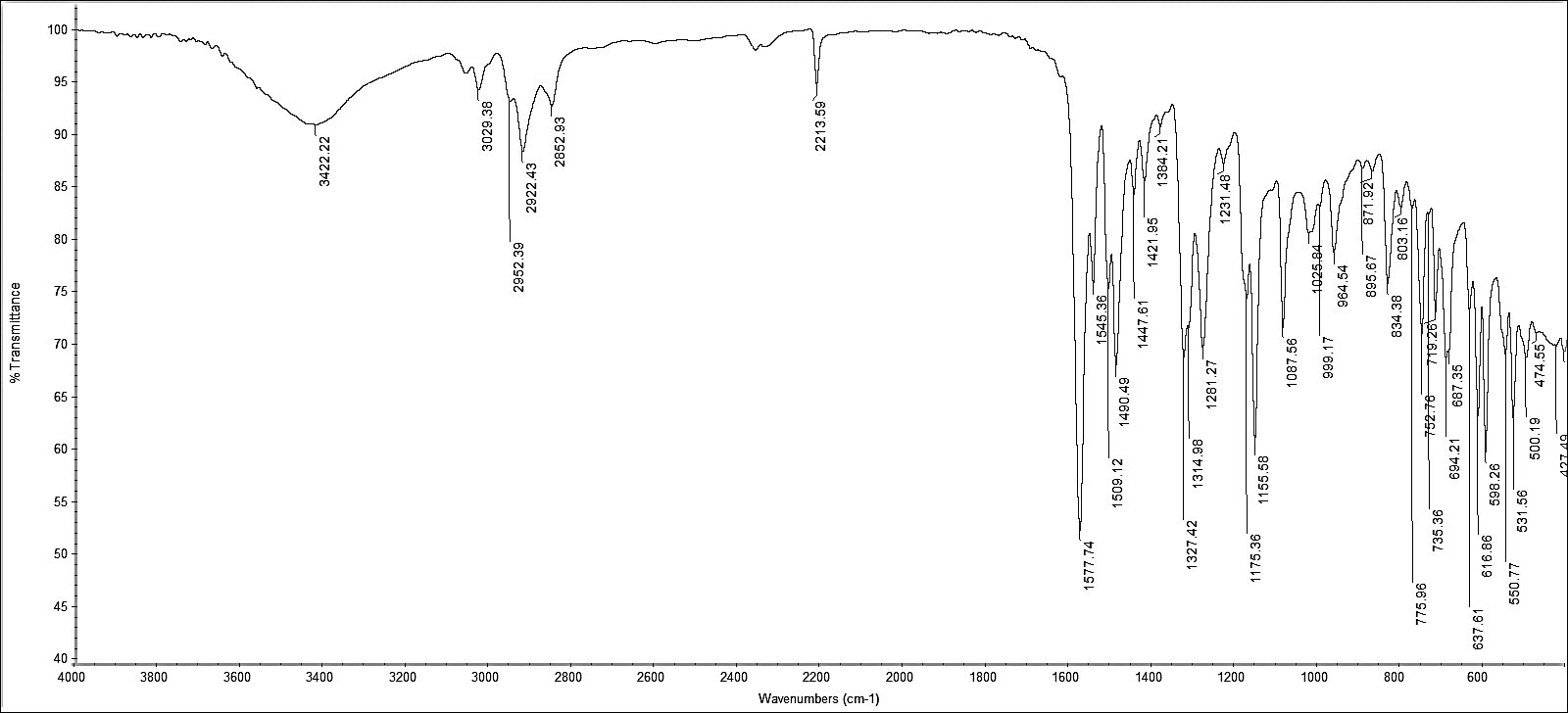
**

**Figure (S17): IR spectrum of dye SAS-3.**

**
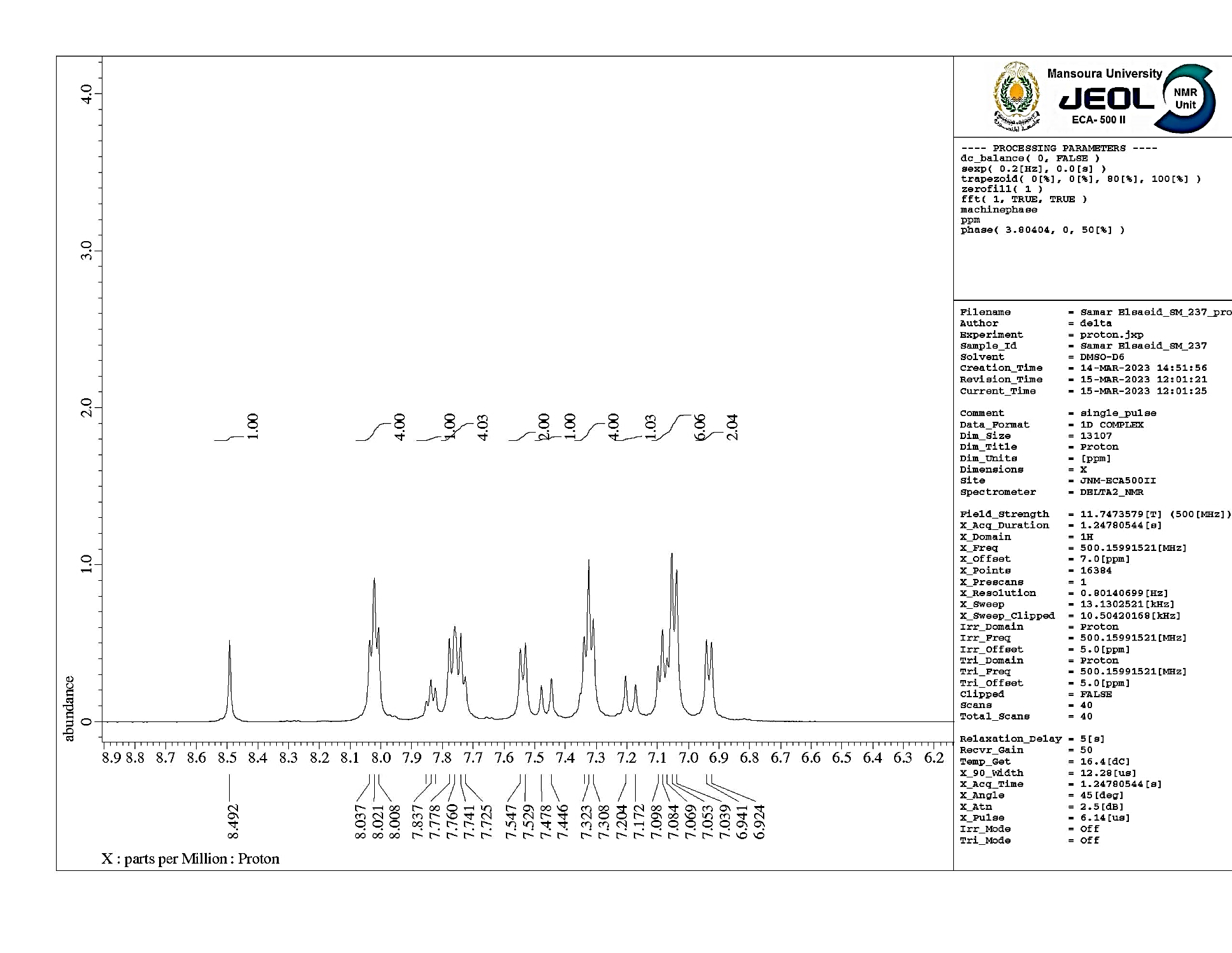
**

**Figure (S18): ^1^H NMR spectrum of dye SAS-3.**

**
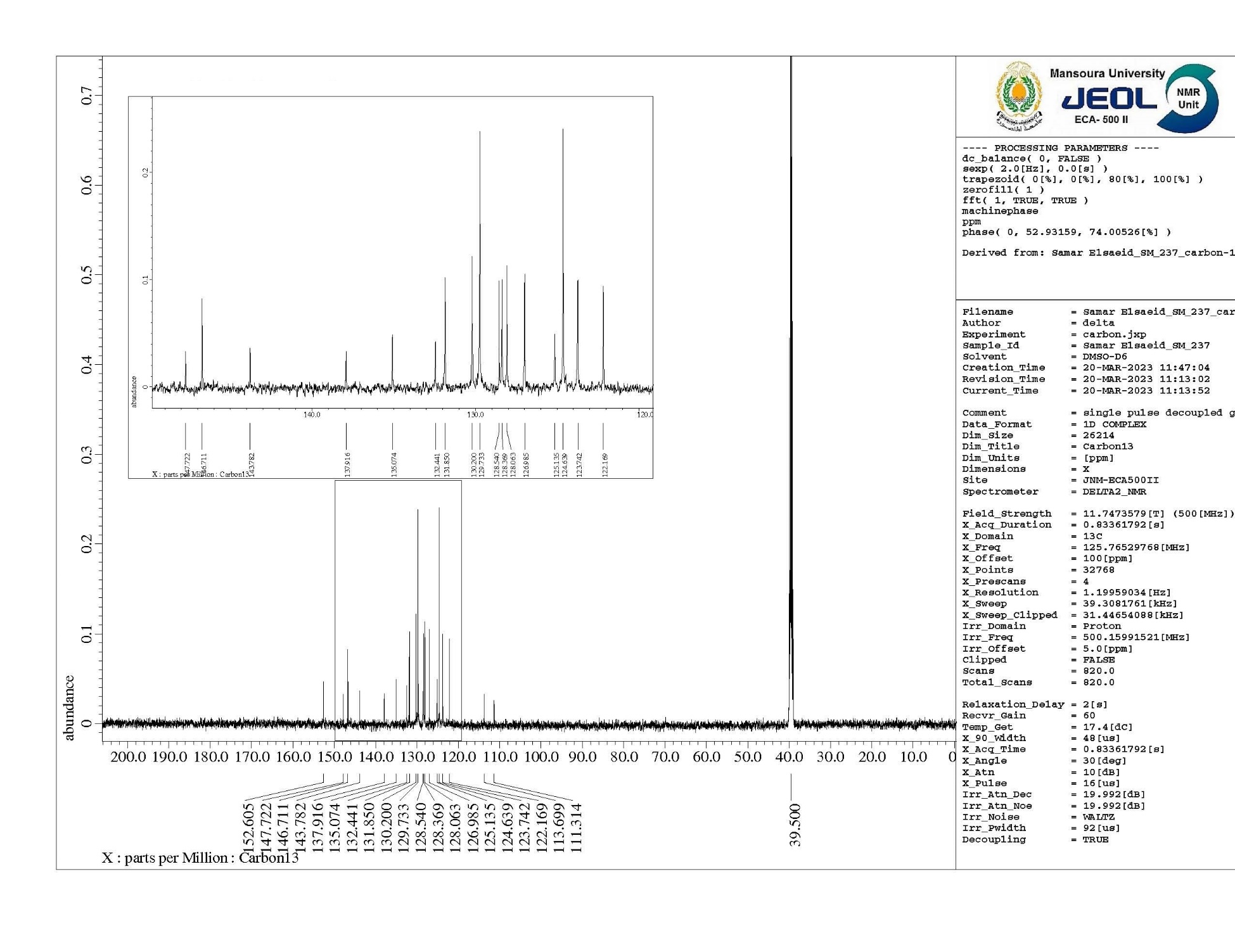
**

**Figure (S19): ^13^C NMR spectrum of dye SAS-3.**

**Figure (S20): Mass spectrum of dye SAS-3.**

**
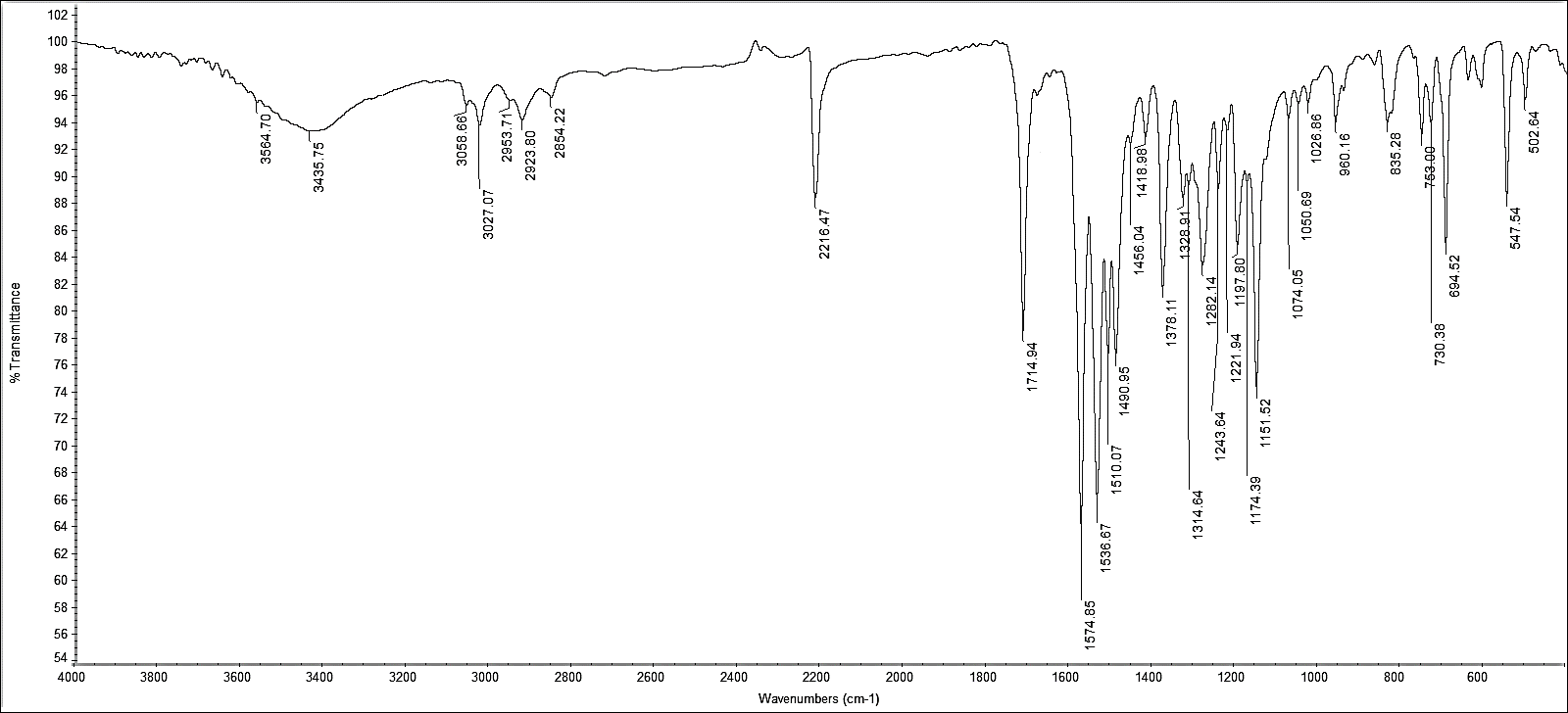
**

**Figure (S21): IR spectrum of dye SAS-4.**

**
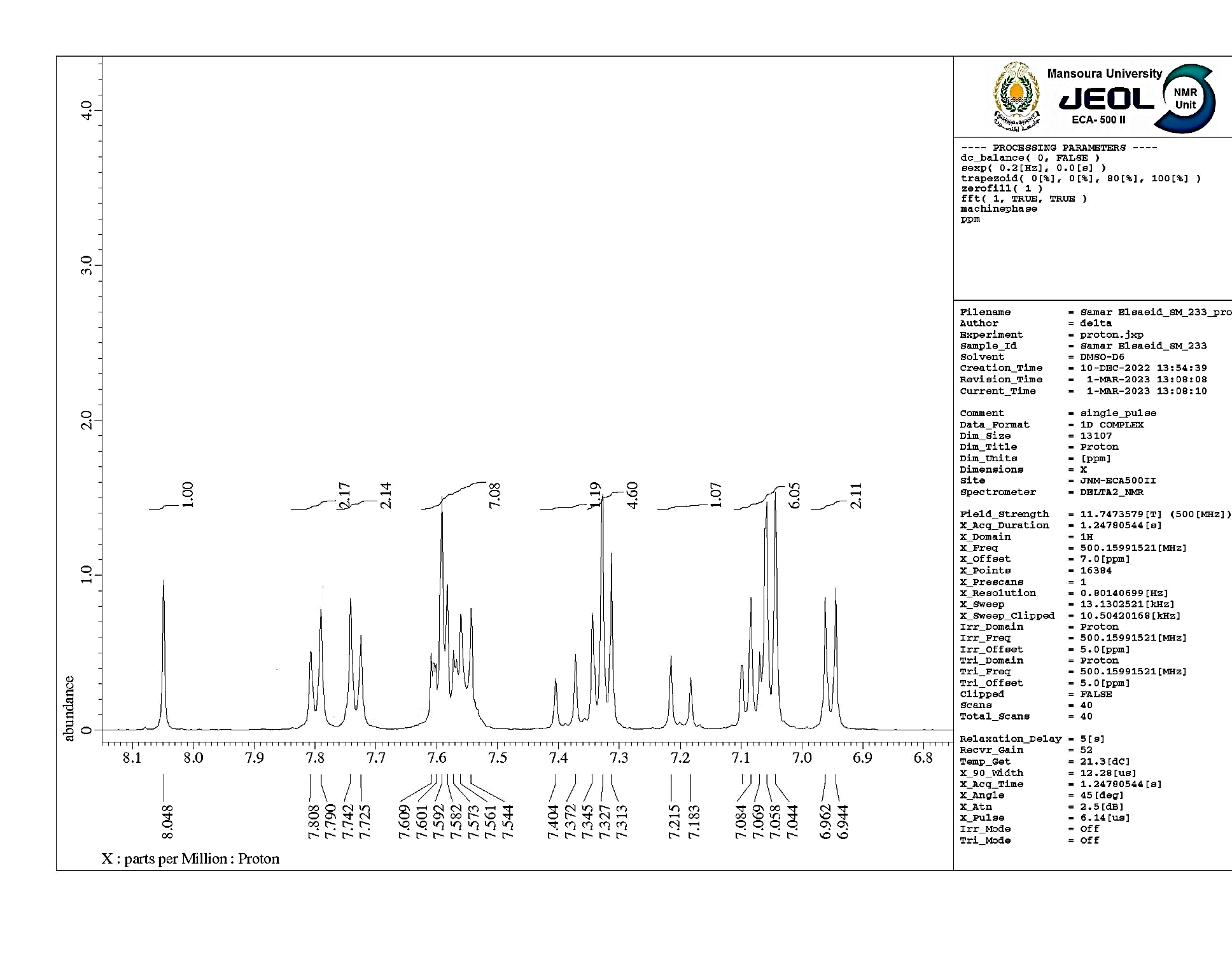
**

**Figure (S22): ^1^H NMR spectrum of dye SAS-4.**

**
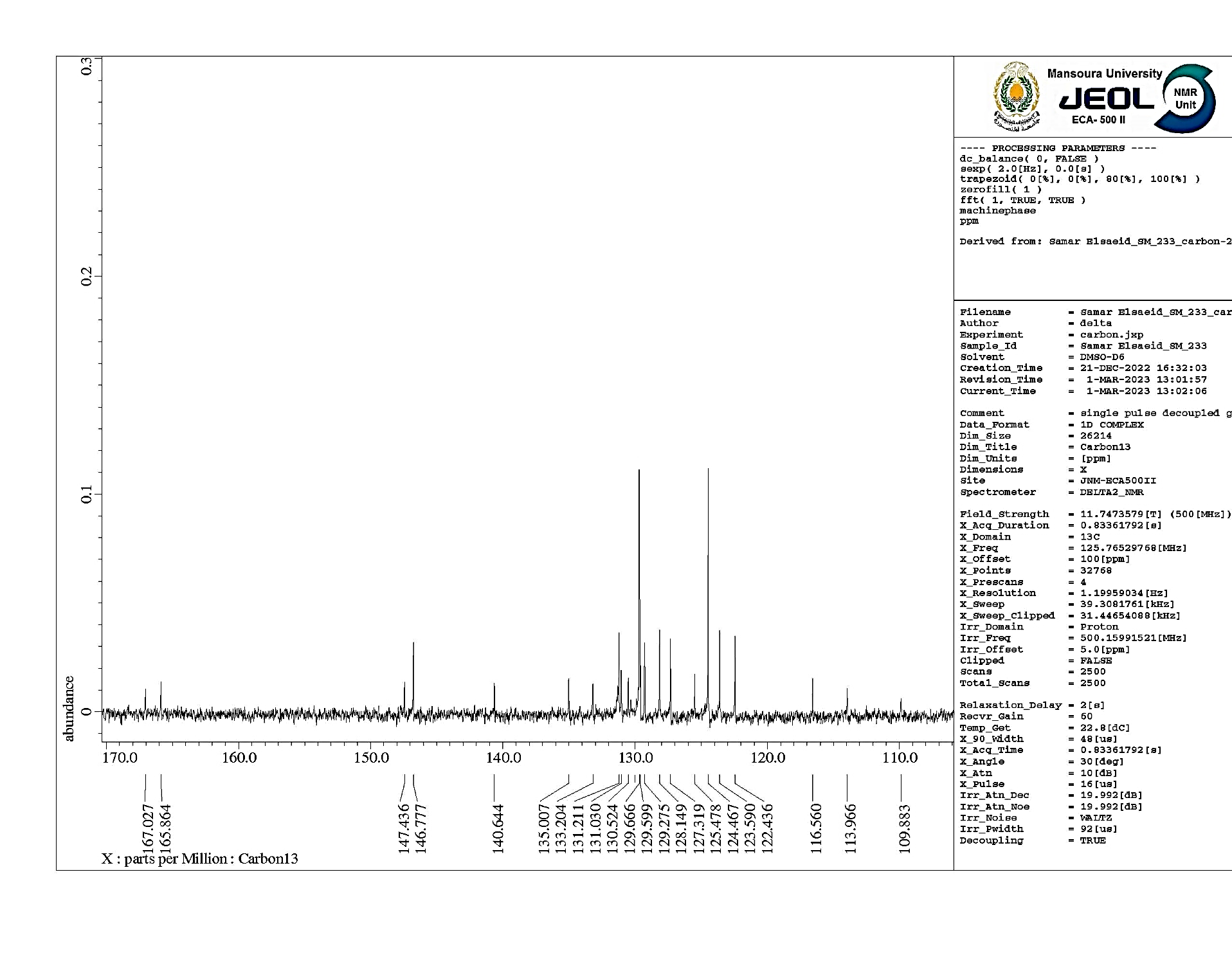
**

**Figure (S23): ^13^C NMR spectrum of dye SAS-4.**

**
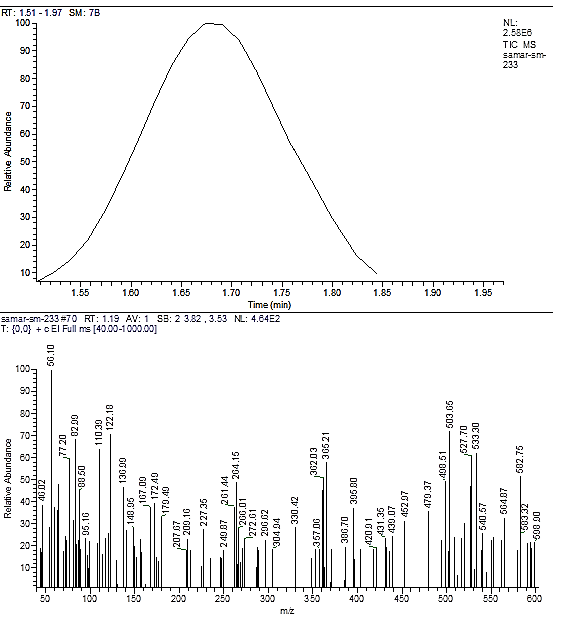
**

**Figure (S24): Mass spectrum of dye SAS-4.**

**
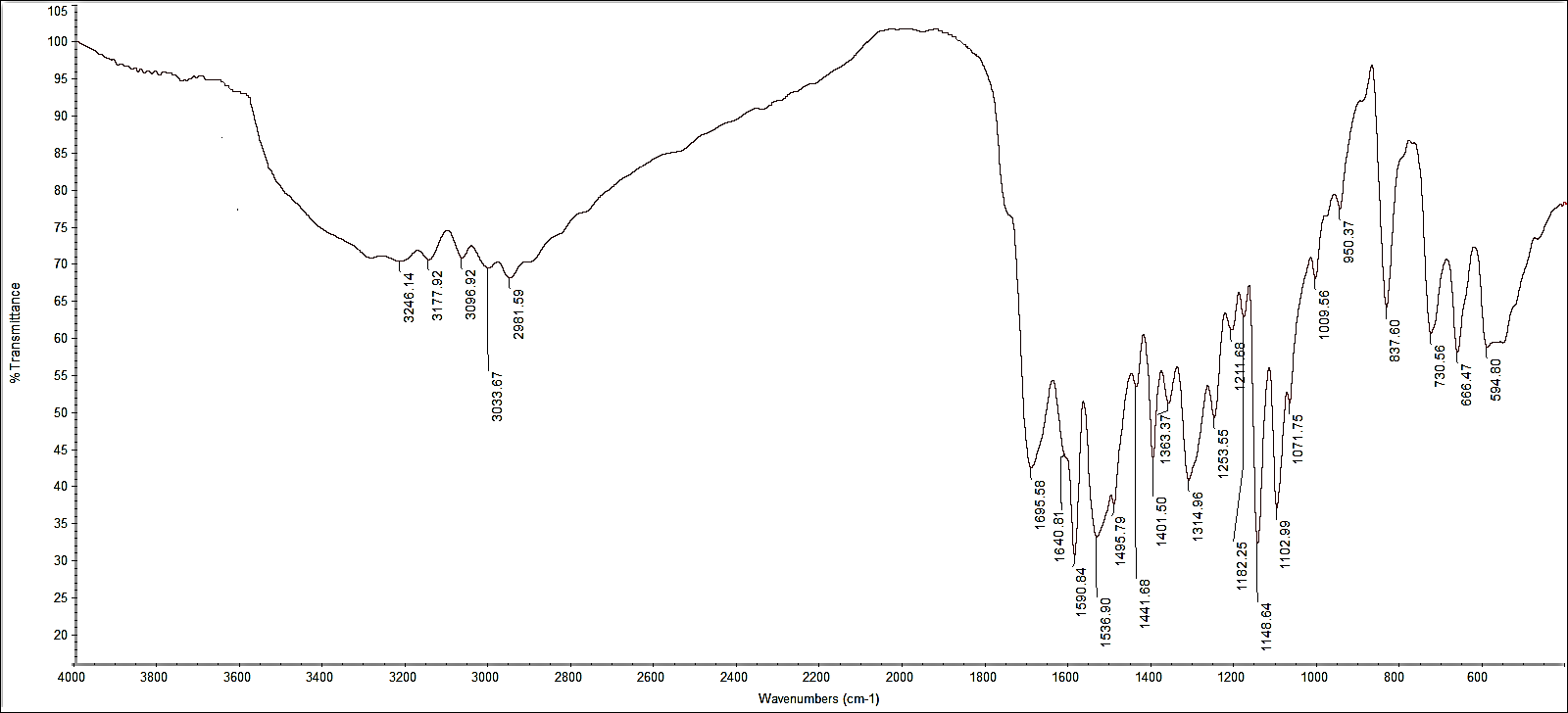
**

**Figure (S25): IR spectrum of dye SAS-5.**

**
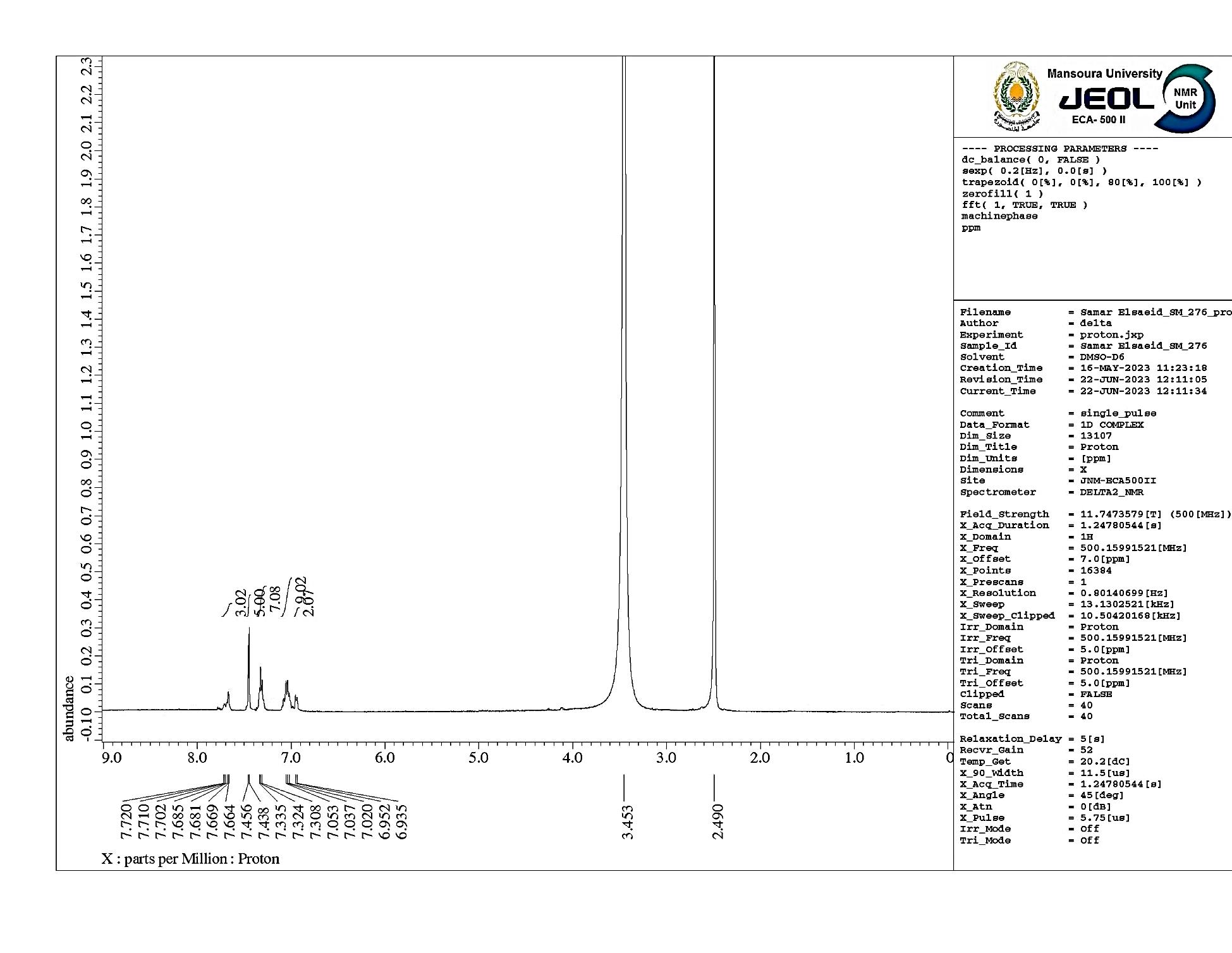
**

**Figure (S26): ^1^H NMR spectrum of dye SAS-5.**

**
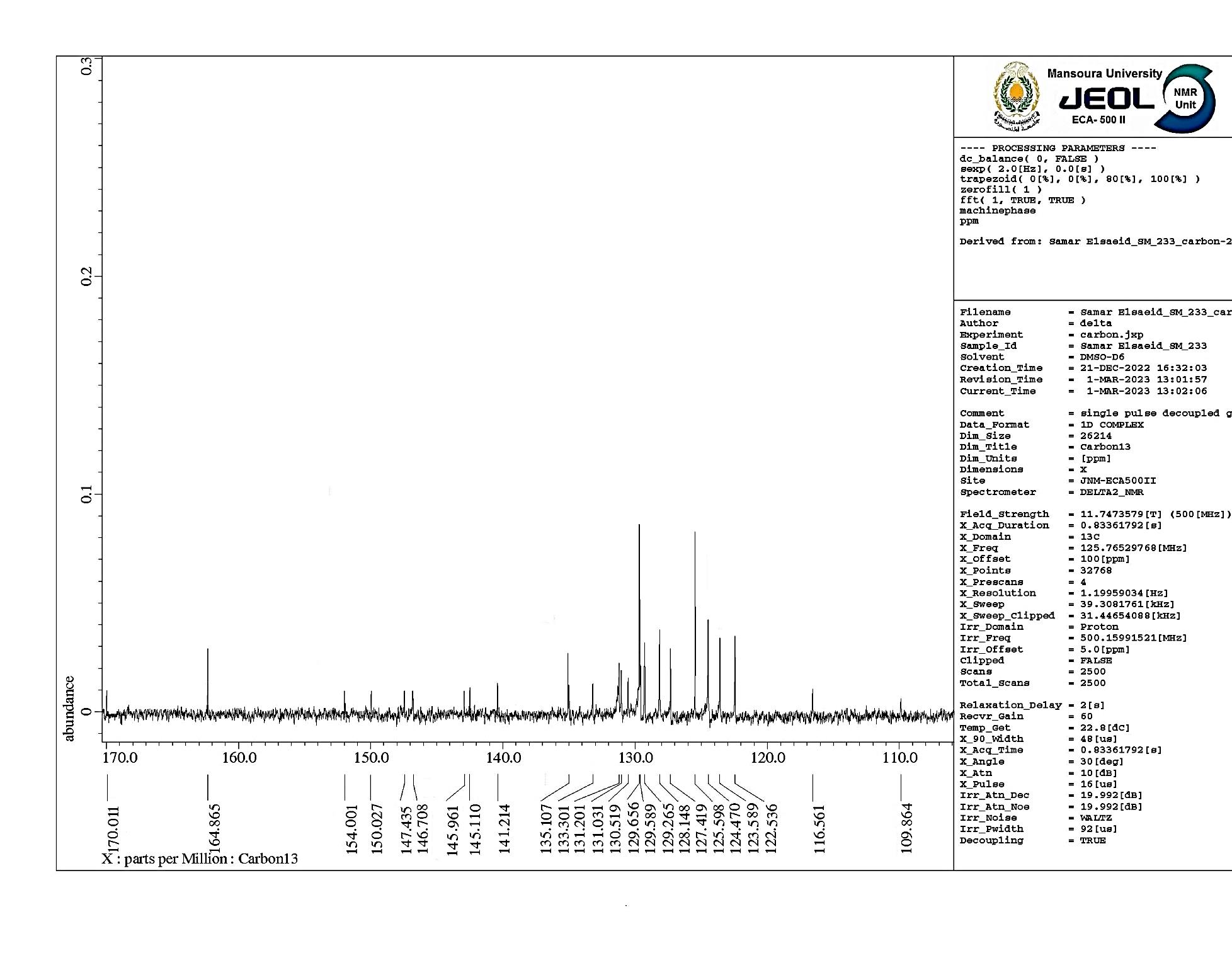
**

**Figure (S27): ^13^C NMR spectrum of dye SAS-5.**

**
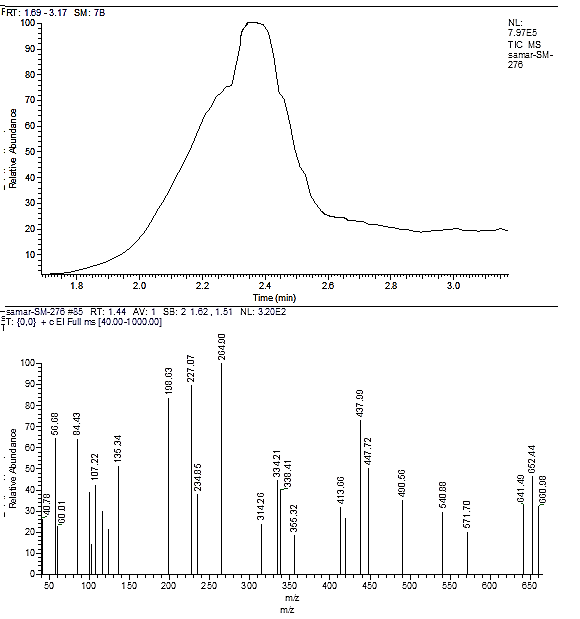
**

**Figure (S28): Mass spectrum of dye SAS-5.**

**
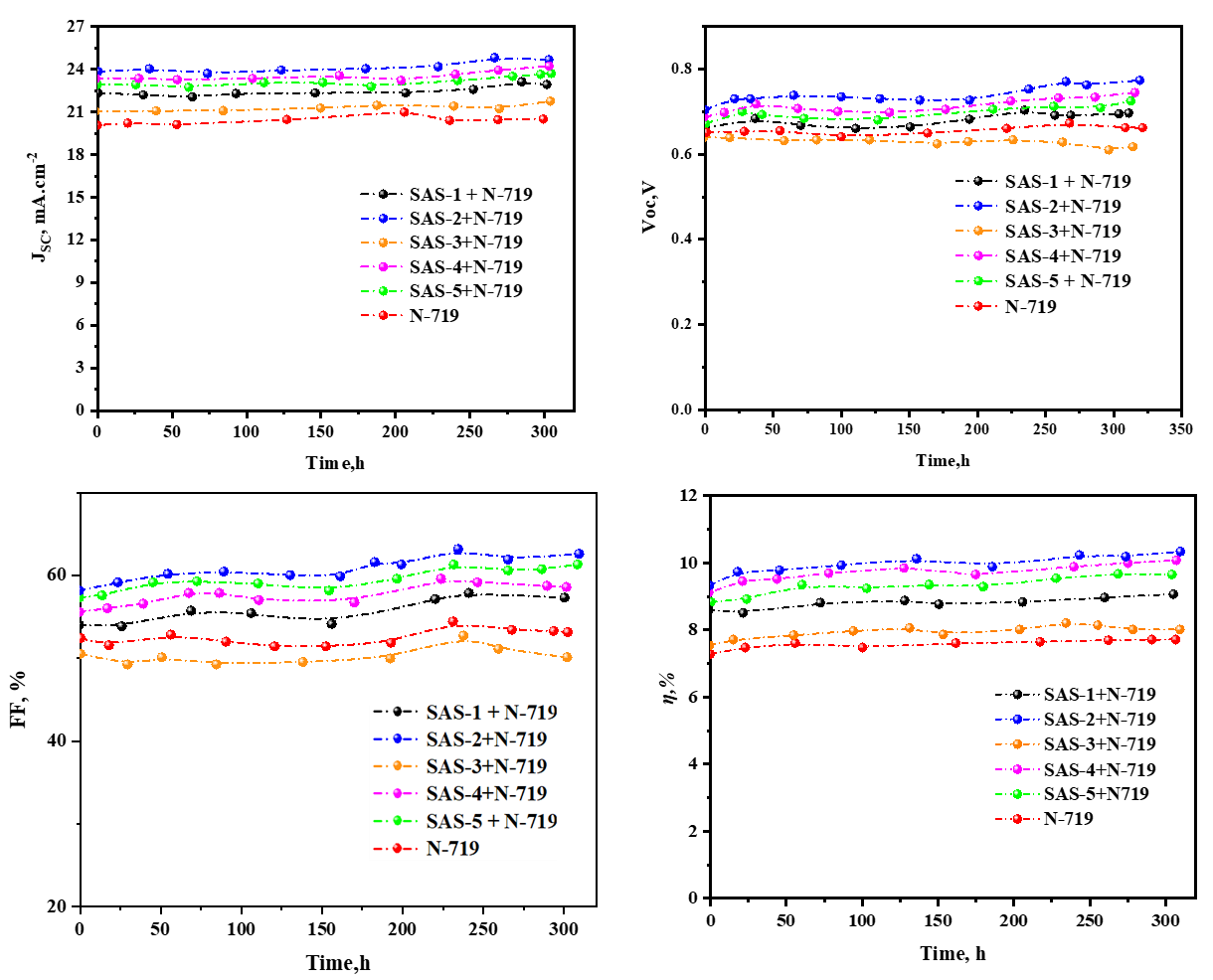
** **Figure (S29). Photovoltaic parameters of the device (SAS-1-5+N719) and N-719 measured under the illumination of sun light for 300 h.**

**Cell preparations and photovoltaic characterizations**

1. **Preparing Electrodes for DSSC**

**(a) Working Electrode (Photoanode-TiO_2_ electrode)**

The fabrication process of dye-sensitized solar cells began with the preparation of a TiO_2_ electrode, which consisted of a double-layer structure with a thickness of 10 + 5 μm. The electrode had a 10 mm thick nano-porous layer and a 5 μm thick scattering layer, which were prepared using a previously reported method [1]. The working electrode is subjected to 15 minutes of sonication in a detergent solution, followed by washing with deionized (DI) water, acetone, and ethanol. The electrode is then treated with TiCl_4_ (60 mM) at 90°C for 60 minutes, and subsequently washed with water and ethanol. A layer of nanoporous TiO_2_ (12-14 μm thick) is printed on the electrode using a single 3M transparent tape. The thickness of the layer is adjusted by punching a hole with a 3/16 5.0 stick. A dispersing layer is printed on top of the TiO_2_ layer using a single 3M transparent tape. The thickness of the layer is adjusted by punching a hole with a 1/4 6.0 stick. The TiO_2_ used for the dispersing layer is R/SP. The printed layers are sintered at 350°C for 10 minutes and then at 500°C for 30 minutes. The electrode is then treated with TiCl_4_ at 90°C for 60 minutes and washed with water and ethanol. The electrode is sintered at 500°C for 30 minutes. In the absence of light, the electrode is submerged in a dye solution for a duration of 20 hours. The organic dye solution is comprised of 0.2 mM of each (SAS-1-5) in a mixture of acetonitrile, tert-butanol, and DMSO (1:1:1) with addition 0.2 Mm N719. Meanwhile, the ruthenium dye solution (N719) consists of 0.2 mM of the dye (SAS-1-5) dissolved in 9 mL solution of consisting of (3 ml) acetonitrile, (3ml) tert-butanol. In the case of co-sensitization, the dye solution consists of a mixture of 0.2 mM of the co-sensitizers (**SAS-1-5**) and 0.2 mM of the ruthenium dye (**N719**) in a 9 mL solution consisting of (3 ml) acetonitrile, (3ml) tert-butanol. The performance of the dye-sensitized solar cells was characterized by photovoltaic measurements of sealed cells were made by illuminating the cell through the conducting glass from the anode side with a solar simulator at AM 1.5 illuminations (light intensity: 100 Mw.cm^−2^)
**The preparation of a counter electrode involves the following steps:**

The electrode is washed with water, followed by a wash with a 0.1M HCl solution in ethanol (0.2 mL of concentrated HCl in 100 mL of ethanol). The electrode is then subjected to 10 minutes of sonication in an acetone bath. The electrode is dried at 400°C for 15 minutes. A layer of Pt-paste is printed on the electrode using a single 3M transparent tape. The thickness of the layer is adjusted by punching a hole with a 3/8 10.0 stick. The Pt-paste used is Platisol T/SP. The printed layer is cured at 450°C for 10 minutes.

[1] Gad, E. A., Kamar, E. M., & Mousa, M. A. (2020). Experimental and computational study on electronic and photovoltaic properties of chromen-2-one-based organic dyes used for dye-sensitized solar cells. *Egyptian Journal of Petroleum*, *29*(2), 203-209.

**2.3. Photovoltaic measurements**

Photovoltaic measurements of sealed cells were made by illuminating the cell through the conducting glass from the anode side with a solar simulator (WXS-155S-10) at AM 1.5 illuminations (light intensity: 100 mW cm^−2^).

**2.4. Incident photon to current efﬁciency (IPCE) conversion**

IPCE measurements were made on a CEP-2000 system (Bunkoh-Keiki Co. Ltd.). IPCE at each wavelength was calculated using Equation 1, where *I_SC_* is the short-circuit photocurrent density (mA. cm^−2^) under monochromatic irradiation, q is the elementary charge, λ is the wavelength of incident radiation in nm and P0 is the incident radiative flux in W/m^2^.

$$\mathrm{IPCE}\left( \lambda\right)=1240\left( \frac{I_{\mathrm{SC}}}{q\lambda P_{o}} \right) (\mathbf{1})$$

**2.5. Electrochemical impedance spectroscopy (EIS)**

The electrochemical impedance spectra were measured with an impedance analyzer potentiostat (Bio-Logic SP-150) under illumination using a solar simulator (SOL3A, Oriel) equipped with a 450 W xenon lamp (91160, Oriel). EIS spectra were recorded over a frequency range of 100 mHz to 200 kHz at room temperature. The applied bias voltage was set at the *V_OC_* of the DSSCs, with AC amplitude set at 10 mV. The electrical impedance spectra were fitted using Z-Fit software (Bio-Logic).

**2.6. Cyclic voltammetry**

Cyclic voltammetry (CV) was performed in DMF with the electrolyte 0.1 M [TBA][PF_6_] at a scan rate of 50 mV s^−1^. The working electrode used is the Glassy carbon, Pt wire represented the counter electrode and the reference electrode is Ag/Ag^+^ in ACN. Fc/Fc^+^ was introduced as internal reference.

**3. Molecular Modeling**

Equilibrium molecular geometries of **SAS-1-5** calculated using the Becke's three parameter hybrid functional, Lee–Yang–Parr's gradient corrected correlation functional (B3LYP) and (6-311g(d, p)) [1, 2, 3, 4]. The geometry optimization calculations were followed by energy calculations using time-dependent density functional theory (TD-DFT) utilizing the energy, functional B3lyp and the basis set 6-311g (d, p). The solvent (DMF) effect was accounted for by using the conductor-like polarizable continuum model (C-PCM), implemented in Gaussian 09.

**References**

[1] G. Melikian, F. Rouessac, C. Alexandre, Synth Commun 23 (1993) 2631.

[2] A. D. Becke, Phys. Rev. A 38 (1988) 3098.

[3] C. T. Lee, W.T. Yang, R.G. Parr, Phys. Rev. B. 37 (1988) 785.

[4] N. Godbout, D.R. Salahub, J. Andzelm, E. Wimmer. Can. J. Chem.-Rev. Can. Chim. 70 (1992) 560-571.
